# Innate immune response to AAV-based gene therapy vectors: Mechanisms of complement activation and cytokine release

**DOI:** 10.1016/j.omtm.2025.101551

**Published:** 2025-08-12

**Authors:** Rebecca Xicluna, Petra C. Schwalie, Emma Bell, Desiree Von Tell, Guido Steiner, Emily Seeger, Julian J. Freen-van Heeren, Annelies W. Turksma, Richard B. Pouw, Timo Schwandt, Michael B. Otteneder, Theo Rispens, Mieke C. Brouwer, Cristina Bertinetti-Lapatki, Hélène Haegel

**Affiliations:** 1 Roche Pharma Research and Early Development, Roche Innovation Center Basel, 4070 Basel, Switzerland; 2 R&D, Sanquin Diagnostic Services, 1066 Amsterdam, the Netherlands; 3 Sanquin Research and Landsteiner Laboratory, Amsterdam UMC, University of Amsterdam, 1066 Amsterdam, the Netherlands; 4 Amsterdam Institute for Immunology and Infectious Diseases, 1105 Amsterdam, the Netherlands

**Keywords:** rAAV, innate immunity, cytokine release, complement activation, AAV capsid, AAV genome, gene therapy

## Abstract

Recombinant adeno-associated viruses (rAAV) have emerged as a preferred strategy for *in vivo* gene delivery. However, the immune response to rAAV presents a major limitation, leading to serious adverse events in clinical trials. This study investigates the interaction between rAAV and the innate immune system. A whole blood assay (WBA) was used to assess complement activation and cytokine release upon stimulation with rAAV in 20 healthy blood donors. Results demonstrate that AAV2 and AAV8 capsids can activate the complement system, primarily through the antibody-dependent classical pathway. Complement activation also occurred in some of the seronegative donors showing the contribution of the alternative pathway. In this WBA setting, there were significant increases in the release of various cytokines and chemokines, with monocytes, natural killer cells, T cells, and B cells identified as the responding cell types using transcriptomics. Cytokine and/or chemokine release was more prominently observed with AAV2 compared with AAV8 and was enhanced by the presence of pre-existing anti-capsid antibodies. Interferon-α release appeared directly dependent on the cytosine-phosphate-guanine (CpG) content of the vector genome. These findings underscore the effects of innate and adaptive immunity to rAAV capsid and genome on the activation of complement pathways and release of inflammatory mediators.

## Graphical abstract

**Figure:**
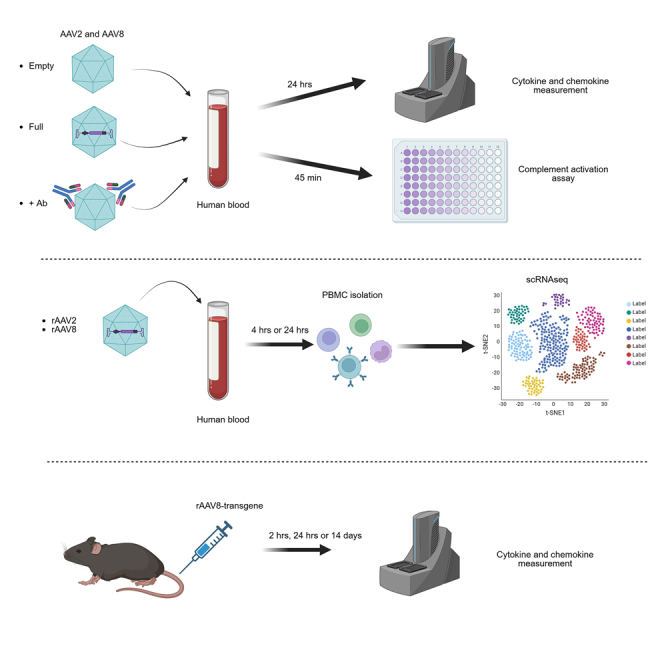

## Teaser

Xicluna and colleagues investigate the mechanisms of cytokine and chemokine release and complement activation in response to rAAV. Using human whole blood assays, they dissect the effects of rAAV capsid and genome on immune cell populations and highlight the influence of pre-existing anti-AAV antibodies and of the genome CpG content.

## Introduction

*In vivo* gene therapy with recombinant adeno-associated viruses (rAAVs) is a promising tool to treat many diseases, as proven by the recent approvals of Zolgensma (onasemnogene abeparvovec), Roctavian (valoctocogene roxaparvovec), and Elevidys (delandistrogene moxeparvovec-rokl)^1^^,^^2^^,^^3^ and the increasing number of clinical trials. AAVs are dependoviruses belonging to the family of Parvoviridae and have not been associated with any disease in humans.^4^^,^^5^ Their safety and low toxicity as well as their ability to infect a large panel of cell types and non-dividing cells have made them a powerful vector candidate. However, despite their relatively low inflammatory properties, the immune response to AAV capsid remains one of the main challenges for efficacy and safety of rAAV-mediated gene therapy.

A large part of the human population (40%–70%) has been naturally exposed to wild-type AAV.^6^ For this reason, pre-existing immunity is widespread, notably characterized by AAV-specific neutralizing antibodies (NAbs) that can cross-react with rAAV. NAbs reduce or even prevent transduction efficacy, in particular upon systemic administration. Currently, patients with AAV-specific NAbs above a defined cutoff titer are excluded from most of the rAAV-mediated gene therapy clinical trials.^7^ Several strategies including B cell depletion, plasmapheresis, or bacterial endopeptidases^8^ are currently developed to lower Ab levels but cannot completely remove them. In addition, rAAV-based gene therapies induce rapid anti-capsid humoral responses that can result in very high antibodies (Abs) titers, which not only prevent re-dosing but may also contribute to adverse events. Therefore, it is important to understand how anti-AAV Abs can impact the overall immune response to treatment.

Another important component of the immune response to rAAV is the adaptive T cell response. Cytotoxic T cell responses to AAV capsid were described for the first time in a clinical trial for Hemophilia B, where the loss of transgene expression was correlated with a transient and asymptomatic elevation of liver transaminases and interferon-γ (IFN-γ) release from peripheral blood mononuclear cells (PBMCs), as detected by IFN-γ enzyme-linked immunospot (ELISpot). The conclusion was that following rAAV dosing, capsid-specific CD8^+^ memory T cells were reactivated leading to the destruction of transduced hepatocytes.^9^ This phenomenon has not been observed in animal models.^10^ In the clinic, cytotoxic T cell (CTL) responses are monitored by IFN-γ ELISpot, but these are not always predictive of clinical outcome, probably due to the scarcity of anti-AAV CD8^+^ T cells in the systemic compartment.^11^ Moreover, this pre-existing cellular response does not seem to correlate with the pre-existing humoral response to rAAV.^12^

The last few years have seen a growing interest in understanding the innate immune response to rAAV. Recombinant AAV vectors have been shown to induce a rapid (within hours) and transient increase in transcripts encoding cytokines and chemokines in the mouse liver.^13^ Cytokines and chemokines were also detected in the plasma or serum of mice and non-human primates (NHP) during the week following intravenous (i.v.) injection of an AAV8 or 9 vector.^14^^,^^15^^,^^16^ Innate immunity not only mediates the first response to the vector, which may cause severe adverse events (SAEs) after systemic administration, but also primes the adaptive immunity.^17^^,^^18^ A study from Kuranda et al. showed that anti-AAV Ab production, which limits the ability to re-dose, is dependent on interleukin (IL)-6 and IL-1β *in vitro* and *in vivo*.^19^ Sensing of the vector genome through Toll-like receptor 9 (TLR9) has been shown to induce type-1 IFN release by plasmacytoid dendritic cells (pDCs) and inhibition of IFN-ɑ production to prevent induction of capsid-specific CD8^+^ T cells.^20^^,^^21^^,^^22^ Furthermore, TLR2 has also been implicated in the sensing of rAAV through capsid recognition.^23^

Complement activation is an important player of the innate immune response. Recently, several gene therapy trials administering high rAAV doses systemically reported SAEs due to complement activation. Within a few days to 2 weeks post-AAV vector infusion, complement activation was associated with thrombotic microangiopathy (TMA).^24^^,^^25^^,^^26^ Complement inhibitors such as the C5 cleavage blocker eculizumab have been used to mitigate these toxicities and the use of the C3 inhibitor APL-9 is currently investigated.^25^^,^^27^ Complement activation can occur through three different pathways: the antibody-dependent classical pathway, the alternative pathway, and the lectin pathway^28^ and can lead to inflammation, opsonization, phagocytosis, and neutralization of the virus and finally results in activation of the adaptive immune response. Complement components have been shown to bind directly to the AAV capsid, inducing complement activation, increasing cytokine and chemokine gene expression, as well as vector uptake in monocytic cell lines, murine bone marrow macrophages, and blood phagocytes and dendritic cells.^13^^,^^27^^,^^29^^,^^30^

This study aimed to further investigate the mechanisms underlying the innate immune response to rAAVs. We used whole blood samples from a panel of healthy donors to analyze the complement activation and cytokine release elicited by rAAV serotypes 2 and 8. These serotypes have been utilized in clinical trials and form the basis of many engineered capsids. Our results in whole blood assays show that the presence of anti-AAV Abs can enhance complement activation and stimulate the release of pro-inflammatory cytokines and chemokines. We also show that IFN-ɑ release is mediated by the AAV genome and reflects its cytosine-phosphate-guanine (CpG) content. Using single-cell RNA-sequencing (scRNA-seq), we identified the main immune cell types involved in this cytokine release. Our results highlight the influence of pre-existing anti-AAV Abs on the immune response to rAAV.

## Results

### AAV capsids can activate the complement cascade through both the classical and alternative pathways

**Figure 1:**
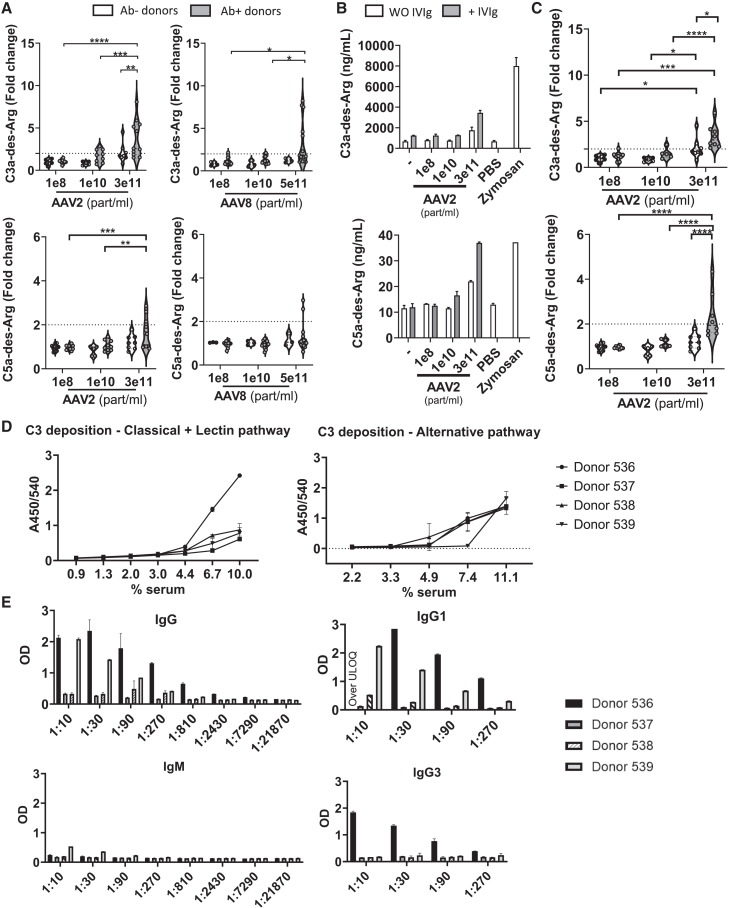
Activation of the complement by AAV2 is dose- and Ab-dependent Whole blood from 20 healthy donors was incubated with different concentrations of AAV2 or AAV8 empty capsids. After 45 min, supernatants were collected and analyzed by ELISA. (A) Fold changes over control (PBS) of C3a-des-Arg and C5a-des-Arg concentrations are shown for 19 of the 20 AAV-seronegative (white plots) or seropositive (gray plots) donors. Dotted lines indicate a 2-fold increase. Data from four independent experiments. (B) Increases in C3a-des-Arg and C5a-des-Arg in whole blood from a seropositive donor incubated with AAV2 in the absence (white bar) or presence (gray bar) of IVIg. (C) C3a-des-Arg and C5a-des-Arg fold-change over control (PBS or PBS+IVIg) in whole blood from *n* = 8 AAV2-seronegative tested without (white) or with addition of IVIg (gray) as surrogate for anti-AAV Abs. Data were from four independent experiments. Data are representative of 12 healthy donors in four experiments. Mean ± SD. Significance was determined with a two-way ANOVA with Holm-Sidak’s multiple comparisons test, ∗*p* ≤ 0.05, ∗∗*p* ≤ 0.01, ∗∗∗*p* ≤ 0.001, ∗∗∗∗*p* ≤ 0.0001. (D) Serum samples from four additional donors were tested for C3 deposition on AAV2-coated plates in buffers that allow activation of either the classical and lectin pathways, or the alternative pathway. (E) IgG, IgM, IgG1, and IgG3 were measured by ELISA in these four donors. IVIg: intravenous immune globulin.

The three pathways of complement activation (classical, alternative, and lectin pathways) all lead to the proteolytic cleavage of C3 into C3a and C3b. C3a is quickly converted to C3a-des-Arg. Downstream of the complement cascade, C5 is cleaved to generate C5a and C5b. As for C3a, C5a is quickly converted to C5a-des-Arg. To investigate the mechanisms of complement activation by rAAVs, we first measured the production of C3a-des-Arg and C5-des-Arg in 20 fresh human whole blood samples incubated with different concentrations of AAV2 or AAV8 (Figures 1A–C). The serological status of the healthy blood donors was determined by ELISA measuring anti-AAV immunoglobulin (Ig)G and IgM titers. Donors were considered seronegative in the absence of detectable anti-AAV IgG and IgM (Table 1).

**Table 1:** Human whole blood (*n* = 20 healthy donors) was treated with 3e11 vp/mL of empty AAV2 or with 5e11 vp/mL of empty AAV8 capsid for 45 min (complement activation) or 3e11 vg/mL of AAV2-null or 5e11 vg/mL of AAV8-null for 24 h (cytokine release)

		# Donor	Anti-AAV IgG titer	Anti-AAV IgM titer	C3 activation	C5 activation	IP-10	MCP-1	MIP-1α	MIP-1β	IFN-γ	IL-1β	IL-2	IL-6	IL-10	IL-8	TNF-α	IFN- α	IFN-β
**AAV2**	**Sero +**	D322	**1/810**	0	1.53	1.02	**25.1**	**13.0**	**3.8**	**5.0**	**5.3**	1.0^a^	0.8^a^	**12.5**	0.2	0.8	**2.5**	**3.2**^a^	1.0^a^
		D326	0	**1/90**	1.86	1.02	1.4	0.9	0.9	0.8	1.0	1.0^a^	**16.3**^a^	**1.8**	1.2	0.7	0.9	**1.7**^a^	1.0^a^
		D327	**1/10**	0	**5.42**	**2.59**	ND	ND	ND	ND	ND	ND	ND	ND	ND	ND	ND	46.0^a^	**21.6**^a^
		D329	**1/10**	0	**8.07**	**2.15**	0.8	**1.6**	1.4	0.6	1.1	1.0^a^	1.0^a^	**1.9**	1.3^a^	0.9	0.2^a^	0.7^a^	1.0^a^
		D331	**1/270**	0	1.48	0.92	**20.7**	**2.9**	**4.2**	**4.8**	**2.1**	**2.3**	**10.5**^a^	**1.6**	0.7	**2.1**	**1.8**	1.5^a^	**2.2**^a^
		D332	**1/30**	0	**2.22**	0.97	**1.6**	1.0	**1.6**	1.2	0.4	**1.9**^a^	1.0^a^	0.4	0.1	0.7	**19.1**^a^	1.0^a^	1.0^a^
		D334	**1/10**	0	**5.15**	1.02	**12.0**	**4.9**	**1.9**	1.2	**6.6**	1.0^a^	1.0^a^	**7.2**	1.0	0.4	0.6	**25.7**^a^	1.9^a^
		D335	**1/30**	0	**4.63**	**2.86**	**2.4**	**2.2**	1.2	0.9	0.9	**8.8**^a^	1.0^a^	**2.4**	1.0^a^	1.3	**1.8**	0.8^a^	1.0^a^
		D336	**1/810**	0	ND	ND	**13.9**	**4.6**	1.2	2.0	**4.3**	0.2	**19.8**^a^	0.4	**1.5**	0.3	0.7	**32.2**^a^	**6.9**^a^
		D337	**1/270**	0	**2.57**	1.91	**17.7**	**4.1**	1.3	1.1	**22.5**^a^	0.1	**9.5**^a^	**1.5**	1.0^a^	0.4	0.5	1.6^a^	1.5^a^
		D338	**1/10**	0	**5.88**	1.62	**2.6**	**3.0**	1.3	**1.6**	**9.0**	1.0^a^	**80.3**^a^	**4.2**^a^	1.0	1.0	**10.3**	1.0^a^	1.0^a^
		D340	**1/7290**	**1/30**	**2.49**	1.58	**11.5**	**17.4**	**6.9**	**8.8**	**14.8**	1.0^a^	**74.9**^a^	**314.2**^a^	**7.6**^a^	0.8	**10.2**	**108.3**^a^	**106.1**^a^
	**Sero**−	D323	0	0	**2.61**	1.17	1.2	1.3	0.8	0.9	1.0	1.0^a^	**82.6**^a^	0.1	0.4^a^	0.2	0.3	ND	ND
		D324	0	0	**2.06**	1.41	**8.1**	**1.7**	1.1	0.9	**72.5**^a^	0.7^a^	**74.1**^a^	0.9	1.0^a^	1.0	0.7	1.0^a^	1.0^a^
		D325	0	0	1.73	0.97	**78.6**	**2.3**	**2.2**	**12.1**	**5.3**	0.6	**25.9**^a^	**3.5**	0.2	0.3	**1.6**	**115.8**^a^	**77.6**^a^
		D328	0	0	**4.54**	1.45	**14.3**	**7.0**	**2.1**	**1.7**	**22.3**^a^	0.7^a^	**4.9**^a^	**1.6**^a^	0.8^a^	1.3	**3.3**^a^	**3.4**^a^	1.0^a^
		D330	0	0	1.70	0.78	**15.6**	**3.9**	1.2	0.7	**2.9**	**6.9**^a^	**5.2**^a^	**3.1**	**3.3**	0.2	**2.4**	**18.0**^a^	1.0^a^
		D333	0	0	0.79	0.91	1.0	1.3	**2.3**	0.8	1.0^a^	1.0^a^	1.0^a^	0.4	1.0^a^	0.3	1.0^a^	1.0^a^	1.0^a^
		D339	0	0	1.56	1.78	**1.6**	1.3	0.8	0.8	0.7	1.0^a^	1.0^a^	0.8	0.8	0.5	0.1	1.4^a^	1.0^a^
		D341	0	0	1.51	1.18	**1.7**	1.3	1.2	0.9	0.9	1.0^a^	1.0^a^	1.4	1.0^a^	0.7	0.3	1.0^a^	1.0^a^
**AAV8**	**Sero +**	D322	**1/810**	0	1.07	0.88	ND	ND	ND	ND	ND	ND	ND	ND	ND	ND	ND	ND	ND
		D323	**1/90**	0	1.70	1.05	ND	ND	ND	ND	ND	ND	ND	ND	ND	ND	ND	ND	ND
		D325	**1/810**	0	1.05	0.93	**237.3**	**1.74**	**1.93**	**3.32**	**5.25**	0.43	**19.16**^a^	**1.72**	1.15	0.28	**1.57**	**71.61**^a^	**43.48**^a^
		D326	0	**1/810**	1.12	1.03	1.15	1.02	0.98	0.95	1.05	1^a^	1^a^	1.23	0.32	0.71	1.06	1.07^a^	1.00^a^
		D327	**1/90**	0	**7.70**	**2.97**	ND	ND	ND	ND	ND	ND	ND	ND	ND	ND	ND	ND	ND
		D328	0	**1/30**	**7.50**	1.50	**7.15**	**5.43**	1.45	1.15	1.15^a^	1^a^	1^a^	**1.51**^a^	0.53^a^	1.20	0.72^a^	**2.13**^a^	1.00^a^
		D329	0	**1/10**	**8.24**	1.32	0.87	1.46	1.38	0.44	1.12	1^a^	1^a^	0.82	1^a^	1.30	0.21^a^	0.48^a^	1.00^a^
		D330	0	**1/90**	1.24	0.61	**7.33**	**1.77**	0.94	0.56	**1.65**	1^a^	1^a^	0.74	0.69	0.56	0.11	**6.61**^a^	1.00^a^
		D331	**1/90**	**1/810**	1.12	0.82	**44.97**	**4.17**	**2.63**	3.6	**2.73**	0.62	1^a^	**2.03**	0.59	0.79	1.40	**2.75**^a^	1.00^a^
		D332	**1/90**	0	**4.61**	1.14	1.41	0.36	1.16	0.87	0.86	0.13^a^	1^a^	0.17	0.1	0.28	0.04^a^	0.52^a^	1.00^a^
		D335	**1/90**	0	**4.12**	**2.58**	1.15	1.18	0.86	0.72	1.1	0.3^a^	1^a^	0.97	1^a^	0.94	0.74	1.00^a^	1.00^a^
		D336	**1/90**	0	ND	ND	0.88	0.59	0.76	0.89	0.6	0.79	1^a^	1.2	2.52	0.64	0.48	1.00^a^	1.00^a^
		D337	**1/270**	0	1.80	1.13	0.82	0.66	0.82	0.85	0.74^a^	0.08	1^a^	0.84	1^a^	0.65	1.27	1.00^a^	0.57^a^
		D338	**1/90**	0	1.63	1.08	0.78	0.68	1.38	1.08	0.94	1^a^	1^a^	1^a^	0.44	0.69	**4.11**	1.00^a^	1.00^a^
		D339	**1/270**	0	**1.70**	0.99	**2.87**	**2.04**	0.9	0.93	**1.71**	1^a^	1^a^	**2.13**	0.83	0.36	1.22	**7.52**^a^	1.00^a^
		D340	**1/90**	**1/270**	**2.31**	0.89	**6.49**	**5.91**	**3.05**	1.49	**2.65**	1^a^	1^a^	**35.38**^a^	1^a^	0.93	**4.36**	**4.57**^a^	1.00^a^
		D341	**1/90**	0	1.66	1.01	0.66	0.65	1.23	0.52	0.08	1^a^	1^a^	0.77	1^a^	0.72	1.18	1.00^a^	1.00^a^
	**Sero** −	D324	0	0	1.15	1.45	1.27	**2.14**	0.85	0.83	0.63^a^	0.7^a^	1.06^a^	1.11	**2.16**^a^	**1.61**	0.25	1.00^a^	1.00^a^
		D333	0	0	1.48	1.05	0.9	0.59	**1.84**	1.4	1^a^	1^a^	1^a^	0.4	1^a^	0.82	1.00^a^	1.00^a^	1.00^a^
		D334	0	0	0.98	0.91	**3.71**	**1.99**	1.07	0.94	**2.04**	1^a^	1^a^	**1.64**	1	0.61	0.65	**4.62**^a^	0.68^a^

Anti-AAV Ab titers were measured by ELISA. Cytokines, chemokines, interferon, C3a-des-Arg, and C5a-des-Arg were measured in the supernatants. The results are indicated in fold increase over PBS control, or over the lower limit of quantification (LLOQ) when concentrations were below the LLOQ in the PBS control. Anti-AAV Ab-positive donors are indicated by Sero+ and anti-AAV Ab-negative donors are indicated by Sero−. Numbers in bold: positive response (Complement activation: ⩾2; Cytokine: ⩾1.5), ND: not determined.

a Indicates the fold increases that were calculated using the LLOQ as value for the PBS control.

By comparing the effects of similar quantities of full (genome-containing) vs. empty capsids in six of the blood donors (Figure S1), we found that the presence of the AAV genome did not impact complement activation, as reported by Smith et al.^27^ The following study performed in 20 healthy blood donors was therefore conducted with empty AAV2 and AAV8 capsids. Interpretable results were obtained for 19 of the 20 donors. Dose-dependent complement activation was observed with both AAV serotypes (Figures 1A and 1B). AAV2 significantly activated complement in a majority of seropositive donors, as shown by C3a-des-Arg and C5a-des-Arg elevation at the highest AAV2 dose (3e11 part/mL) (Table 1). However, no correlation was observed between the anti-AAV Ab titers and the increases in C3a-des-Arg or C5a-des-Arg. To further assess whether complement activation was Ab-dependent, intravenous immunoglobulins (IVIgs) containing anti-AAV2 IgGs (Figure S2) were used as a surrogate for natural anti-AAV Abs and added to the blood of seronegative donors (Figures 1B and 1C). At the highest concentration of AAV2, there were significant increases in C3-des-Arg and C5-des-Arg when IVIg was added. Interestingly, three of the seronegative donors showed increases in C3a-des-Arg in response to AAV2 and AAV8 in the absence of detectable anti-AAV IgG or IgM (Table 1). These results suggested a contribution of the alternative pathway, or of other anti-AAV Ig isotypes.

To further investigate the mechanism of complement activation by AAV2, activation of the complement system on the AAV surface was assessed using pathway-specific ELISAs, using C3 deposition as a readout.^31^ Serum samples from four additional blood donors were incubated on AAV2-coated plates in buffers that allow only specific complement activation routes, i.e., the classical and lectin pathway, or the alternative pathway (Figure 1D). Total anti-AAV2 IgG and IgM as well as IgG1 and IgG3 were measured by ELISA in these four donors (Figure 1E). We chose to focus on the IgG1 and IgG3 isotypes because they are the most effective at activating the classical complement pathway.^32^ The first ELISA was performed in a buffer allowing complement activation through the classical and/or lectin pathways. All tested donor sera could activate these pathways, as shown by C3 deposition on coated IgM (classical pathway) and mannan (lectin pathway) (Figure S3). Incubation of the four donor sera on AAV2-coated plates resulted in concentration-dependent C3 deposition, more pronounced for donor 536. As this donor also had the highest titers of anti-AAV2 IgGs, the increased C3 deposition as compared with the other donors was highly suggestive of classical pathway-mediated complement activation on the AAV surface. Sera of donors 537 and 538, which had little to no detectable anti-AAV2 IgG and IgM, resulted in low C3 deposition on the AAV surface. Strikingly, donor 539, which contained anti-AAV2 IgG and IgM did not show increased C3 deposition as compared with the seronegative donors. Despite his high level of total anti-AAV2 IgG (mainly of the IgG1 subtype) and IgM, this donor had no detectable anti-AAV2 IgG3 in contrast to donor 536, suggesting that presence of IgG3 to the AAV2 capsid might be required to efficiently induce classical pathway activation in this set-up.

C3 deposition also was assessed on the AAV surface in conditions allowing complement activation through the alternative pathway. Sera from all four donors were found to activate the alternative complement pathway, as shown by C3 deposition on coated lipopolysaccharide (LPS) (Figure S3). There were no clear differences between donors. At around 11% (v/v) serum concentration, all donor sera showed C3 deposition. These results show that AAV2 capsids can activate the alternative pathway of complement activation, in addition to the Ab-dependent classical pathway.

### Cytokine/chemokine/IFN release in response to rAAV is enhanced in presence of anti-AAV Abs *in vitro*

To explore the early cytokine and chemokine release triggered by rAAV2 and rAAV8, as well as the impact of anti-capsid Abs, we used a whole blood assay system. Fresh whole blood from healthy donors exhibiting a range of anti-AAV2 and anti-AAV8 IgM and IgG titers (Table 1) was incubated with different concentrations of rAAV2 or rAAV8 “null” vectors (containing genomes devoid of promoter and transgene). Twenty-four hours later, cytokines and chemokines were quantified in the supernatants.

**Figure 2:**
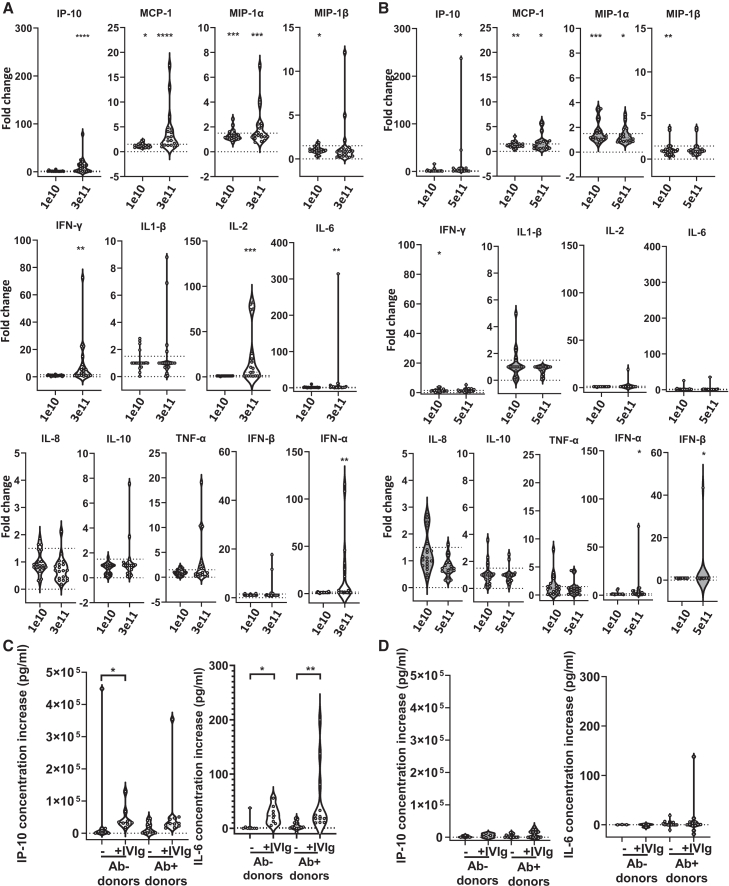
rAAV2 and rAAV8 trigger cytokine, chemokine, and interferon release in whole blood Whole blood from 20 healthy blood donors was incubated with different concentrations of rAAV. After 24 h, supernatants were collected for cytokine and chemokine measurement. Data could be obtained from 19 AAV2-treated and 18 AAV8-treated blood samples, of the 20 donors tested in four independent experiments. (A and B) Cytokine concentration changes upon rAAV2 stimulation (A) or rAAV8 stimulation (B). Fold changes are calculated over the control value, or over the lower limit of quantification (LLOQ) if the control value is 0. Dashed lines correspond to 0 and 1.5-fold increase. Significance was determined by one-sample one-sided Wilcoxon test. (C and D) IP-10 and IL-6 concentration increase over control in seropositive and seronegative donors, in the absence or presence of IVIg and upon stimulation with rAAV2 (C) and rAAV8 (D). IVIg: intravenous immune globulin. Significance was determined by the Kruskal-Wallis test with Dunn’s multiple comparisons test. ∗*p* ≤ 0.05; ∗∗*p* ≤ 0.01; ∗∗∗*p* ≤ 0.001; ∗∗∗∗*p* ≤ 0.0001.

Upon stimulation with rAAV2 or rAAV8 (results from *n* = 19 and *n* = 18 donors, respectively), no changes were observed in IL-4, IL-12p70, IL-13, eotaxin, eotaxin-3, macrophage-derived chemokine (MDC), monocyte chemoattractant protein (MCP)-4, or thymus and activation-regulated chemokine (TARC) concentrations (data not shown). In response to rAAV2, there was an upregulation of several chemokines (IP-10, MCP-1, macrophage inflammatory protein (MIP)-1ɑ, MIP-1β, and IL-8) and inflammatory cytokines (IL-1β, IL-2, IL-6, IL-10, IFN-ɣ, and tumor necrosis factor (TNF)-ɑ) in some donors (Figure 2A). There were significant increases in IP-10, MCP-1, MIP-1ɑ, IFN-ɣ, IL-2, and IL-6 among the cohort. IL-2 and/or IFN-ɣ release was strongly increased in some of the donors, suggestive of pre-existing T cell responses. Type I interferon was also detected in some of the donors (Figure 2A). These increases were significantly rAAV2-dose-dependent for IP-10, MCP-1, IFN-ɣ, IL-2, and IFN-ɑ (Figure S4). Increases in MCP-1, MIP-1ɑ, MIP-1β, IL-6, and TNF-ɑ concentrations were generally stronger in seropositive donors; however, there were no clear correlations between the anti-AAV2 IgG or IgM titers and fold increases in cytokines (Table 1). Additionally, no correlation was observed between complement activation and cytokine or chemokine release (Table 1).

Despite the higher concentration of rAAV8 (5e11 vg/mL vs. 3e11 vg/mL for rAAV2), cytokine release was less pronounced in response to this serotype compared with rAAV2. Still, AAV8-dependent increases in IP-10, MCP-1, MIP-1ɑ, MIP-1β, and IFN-ɣ increases were statistically significant (Figure 2B). The rAAV2 dose-dependency was significant only for IP-10 (Figure S4; Table 1). IP-10 and IFN-ɑ showed stronger increases in some seropositive donors (Table 1), but our cohort including only three AAV8 seronegative donors was too small for a fair comparison.

To further investigate the effects of anti-AAV Abs on cytokine release, we supplemented the whole blood assay with exogenous pooled human immunoglobulins (IVIgs). In the presence of IVIg, all of the donors showed elevations in IP-10 and IL-6 release in response to rAAV2 (Figure 2C). IVIg also enhanced IP-10 and IL-6 release in response to rAAV8 but not in all donors (Figure 2D): this might be at least in part because the anti-AAV8 Ab titer was lower than anti-AAV2 in IVIg (Figure S2). In some donors, IVIg also increased the levels of chemokines MCP-1 and MIP-1β and of interferons IFN-ɣ, IFN-ɑ, or IFN-β in response to both rAAV2 and rAAV8. MIP-1ɑ, TNF-ɑ, or IL-1β release also increased in response to rAAV2 (data not shown).

### The AAV genome stimulates cytokine and chemokine release and the IFN-ɑ response reflects its CpG content

**Figure 3:**
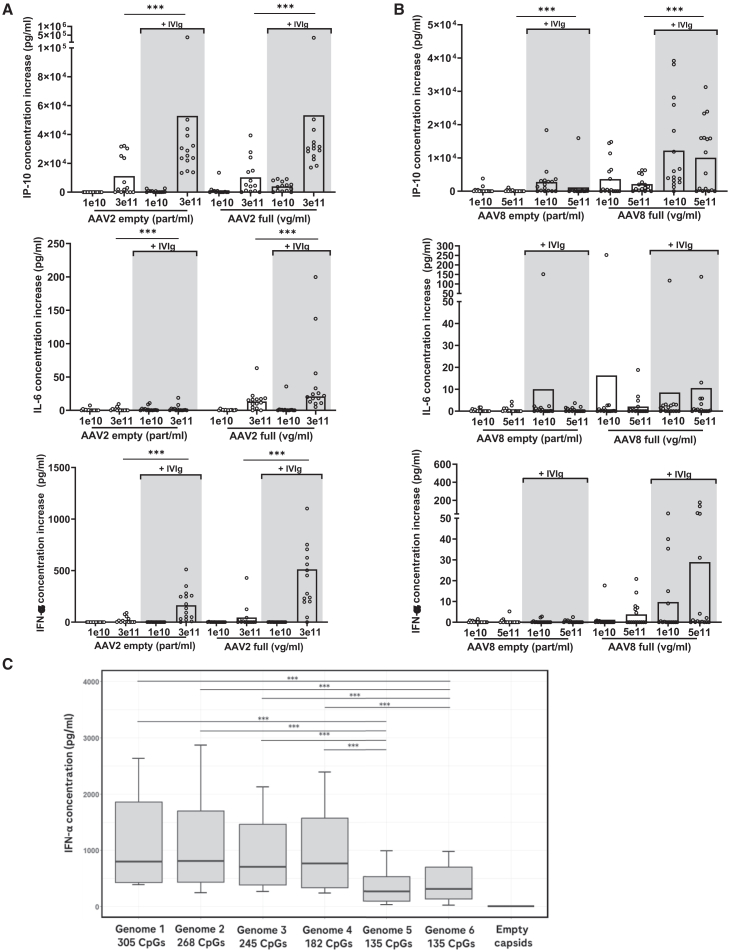
The presence of a genome enhances the cytokine response to AAV Fresh blood from healthy donors of the 20-donor cohort was incubated with different concentrations of rAAV2-null or empty AAV2 capsids (*n* = 14) (A) or with rAAV8-null or empty AAV8 capsids (*n* = 15) (B) in the presence or absence of IVIg. After 24 h, supernatants were collected for cytokine measurements. Concentration increases are shown for IP-10, IL-6, and IFN-α over PBS control. Bars indicate the median. Data from four independent experiments. (C) IFN-α concentration upon stimulation of whole blood from 10 other blood donors with AAV2.7m8 with genomes containing various numbers of CpG motifs in presence of IVIg. All the genome-containing capsids induced statistically significant IFN-α release, compared with the empty capsid (*p* ≤ 0.001). IVIg: intravenous immune globulin. Statistical test described in materials and methods; *p* values non-adjusted. ∗∗∗*p* ≤ 0.001.

IFN type I release has been shown to occur in pDCs recognizing unmethylated CpG motifs in the AAV genome through TLR9 in presence of anti-AAV antibodies.^20^^,^^22^ To further investigate the impact of the genome on cytokine release, fresh blood from healthy donors of the previous cohort was incubated with either genome-containing rAAV2 (*n* = 14) or rAAV8 (*n* = 15) (AAV-null, devoid of promoter and transgene) or with the same amounts of empty capsids. Full:empty capsid ratios of the AAV-null production batches were checked by electronic microscopy and were >90%. The endotoxin levels were below the limit of detection. Addition of IVIg generally increased cytokine release, as previously observed. In the presence of AAV genomes, there were trends to increases in IFN-ɑ release with both AAV serotypes (Figures 3A and 3B). Increases in IL-6 and in IP-10 release were also observed for some of the donors with rAAV2 and rAAV8, respectively. There was generally high donor-dependent variability. Other pro-inflammatory cytokines and chemokines characteristic of the innate immune response like MCP-1 and IL-1β also tended to increase (data not shown). The presence of the genome did not seem to impact the release of IL-2 or IFN-ɣ, markers of T cell and natural killer (NK) cell responses that were not upregulated in the donors tested.

This AAV genome-dependent release was clearly observed in the case of IFN-ɑ, even though it did not reach statistical significance due to the high donor-dependent variability. With AAV8, no IFN-ɑ increase was detected in response to empty capsids while AAV8-null triggered IFN-ɑ release in some of the donors (Figure 3B), particularly visible in the presence of IVIg. To investigate the correlation between IFN-ɑ release and the CpG content of AAV genome, AAV2.7m8 vectors containing transgene expression cassettes with various numbers of CpG motifs were tested in the same whole blood assay in the presence of IVIg (*n* = 10) (Figure 3C). All rAAVs containing genomes triggered significant increases in IFN-ɑ response compared with the empty capsids. The two AAVs containing genome 5 and 6 with the lowest numbers of CpG motifs (135 total CpGs – 0 CpG in the open reading frame [ORF]) triggered significantly lower IFN-ɑ responses compared with the rAAVs with high CpG content: genome 1 (305 CpGs – 170 CpG in the ORF), 2 (268 total CpGs – 133 CpG in the ORF), 3 (245 total CpGs – 110 CpG in the ORF) and 4 (182 total CpGs – 68 CpG in the ORF). These results demonstrate the impact of the CpG motif content of rAAV genomes on interferon type I release, as already shown by others in murine models.^19^^,^^20^ This *in vitro* human whole blood assay system appears as a relevant tool for assessing the potential of rAAV genomes to stimulate the innate immune response.

### Cytokine, chemokine, and IFN release in mice injected i.v. with a high rAAV8 dose

**Figure 4:**
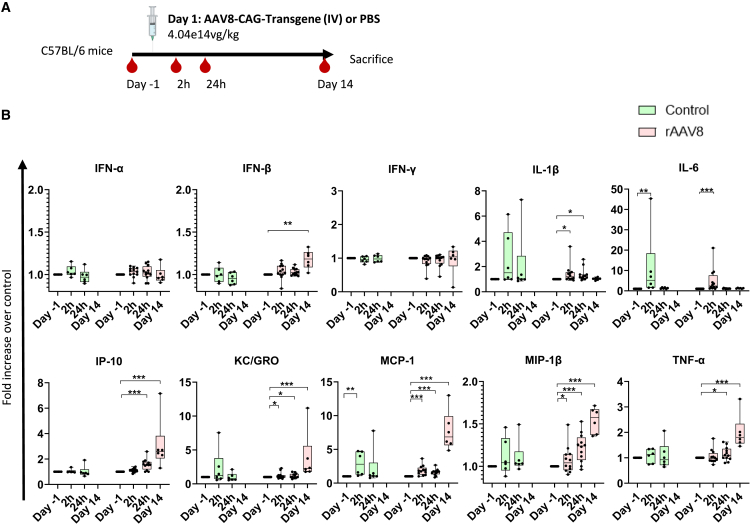
rAAV8 dosing triggers cytokine and chemokine release in mice (A) Mice were injected i.v. with a rAAV8 (4,04e14 vg/kg) or vehicle (control group) (*n* = 10 and *n* = 6, respectively). Serum was sampled 24 h before injection and 2 h, 24 h, and 14 days (only for the rAAV8-treated group) after injection. (B) IP-10, IFN-α, IFN-β, IFN-γ, KC/GRO, IL-1β, IL-6, MCP-1, MIP-1β, and TNFα fold increases over control (pre-dose). Red bars: rAAV8-treated group; green bars: control group. Statistical test described in materials and methods; *p* values non-adjusted. ns: nonsignificant, ∗*p* ≤ 0.05; ∗∗*p* ≤ 0.01; ∗∗∗*p* ≤ 0.001.

We investigated whether some of the cytokines and chemokines identified in human blood stimulated with rAAV were also released *in vivo*. Although our *in vitro* data above suggested that AAV8 was less inflammatory than AAV2, we used AAV8 for this *in vivo* study because this serotype is more commonly used for systemic administration. Mice were injected i.v. with an rAAV8 at the high dose of 4.04e14 vg/kg (Figure 4A). Cytokines, chemokines, and IFNs were measured in the serum sampled before dosing or 2 h, 24 h, and 14 days after dosing.

Treatment with AAV8 induced significant and prolonged increases in several chemokines that were detectable as soon as 2 h after dosing (keratinocyte chemoattractant [KC]/growth-regulated oncogene [GRO], MCP-1, MIP-1β), after 24 h (IP-10) and up to day 14 (Figure 4B). IP-10 remained stable in the control group (mean: 5,382 pg/mL at day −1 and 5,271 pg/mL at 24 h) while it increased in the AAV-treated group (mean: 5,465 pg/mL at day −1 to 7,752 pg/mL at 24 h, and 9,369 pg/mL at day 14). rAAV8 also elicited a statistically significant increase in IL-1β 2 h and 24 h after treatment (mean: 254 pg/mL at day −1, 447 pg/mL at 2 h, 437.28 pg/mL at 24 h), while TNF-ɑ slightly increased 24 h after AAV8 treatment (mean: 40 pg/mL at day −1, 47 pg/mL at 24 h) and remained upregulated until day 14 (mean: 60 pg/mL at day 14). Furthermore, IL-6 was found to increase 2 h after rAAV8 injection (mean:137.5 pg/mL at day −1 and 665pg/mL at 2 h). Unexpectedly, IL-6 also increased in the PBS-treated group (mean: 55.5 pg/mL at day −1 and 983 pg/mL at 2 h). Early and transient increases in IL-1β, MIP-1β, KC/GRO, and MCP-1 were also observed in some of the PBS-treated mice, likely due to the dosing procedure. No increase in circulating IFN-ɣ was observed. For type I IFNs, there was only a modest upregulation of IFN-β detected at day 14, while no significant increase was observed for IFN-α. Cytokines identified in response to rAAV8 both *in vitro* in human blood and in mice were IP-10, MCP-1, and MIP-1β. Some of the cytokines released in human blood in response to rAAV2 were also induced in mice in response to rAAV8: IL-1β, IL-6, and TNF-ɑ. The cytokine and chemokine release may be amplified in mice treated with this very high AAV dose; non-immune cell types as well as tissue-resident immune cells likely contribute to this release.

### rAAV8 induces different transcriptomic profiles in human whole blood

**Figure 5:**
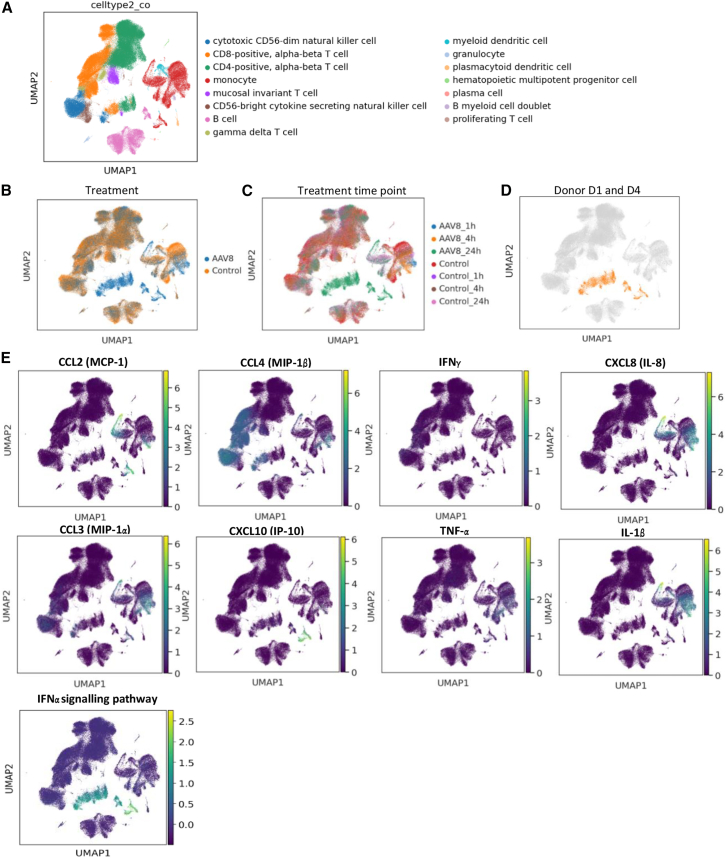
rAAV8 triggers changes in the transcriptome profile in human fresh blood Single-cell RNA-sequencing was performed on human PBMCs isolated from whole blood (*n* = 6) and treated with 5e11 vg/mL of AAV8-null (genome without transgene). UMAP plots of PBMCs colored by cell type (A), treatment (B), and treatment and time point (C). (D) UMAP showing D1 and D4 at 24 h after AAV8-treatment in orange among all other donors, treatments, and time points. (E) UMAP plots showing IFN-α response, CCL2 (MCP-1), CCL3 (MIP-1α), CCL4 (MIP-1β), CXCL8 (IL-8), CXCL10 (IP-10), IL-1β, TNF-α, and IFN-γ gene expression within the cell clusters depicted above.

**Figure 6:**
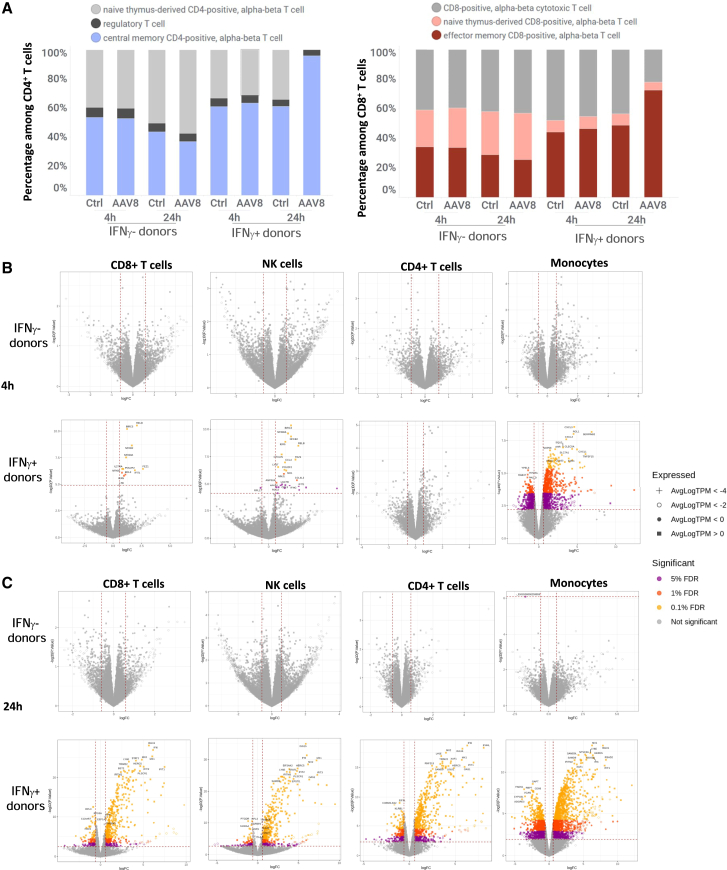
rAAV8 triggers strong transcriptomic changes in blood from highly seropositive, IFN-ɣ producing donors Single-cell RNA-sequencing was performed on human PBMCs isolated from whole blood (*n* = 6) and treated with 5e11 vg/mL of AAV8-null (genome without transgene). (A) Percentages of T cell subtypes found 4 h and 24 h after treatment with AAV8-null in blood from IFN-γ-positive or -negative donors. (B and C) Volcano plots showing the strength of the effect (*x* axis: log fold-change) and its significance (*y* axis: −log10(*p*-value)) for each gene at 4 h (B) and 24 h (C) in IFN-γ negative (left) or -positive donors (right).

We further aimed to explore which immune cell types could be involved in AAV-induced cytokine, chemokine and interferon release. To this end, we conducted single-cell RNA-sequencing (scRNA-seq) of PBMCs purified from whole blood of six new healthy human donors (Table 2), comparing samples taken either before or after 1, 4, or 24 h of incubation with rAAV8. While systemic increases were observed in mice in response to rAAV8, cytokine increases *in vitro* in human blood were limited. We therefore applied transcriptomics to explore the blood cell response to the AAV8 treatment. As a control, scRNA-seq was performed on PBMCs from untreated whole blood incubated under the same culture conditions to consider the potential impact of the *ex vivo* culture on the blood cells. Anti-AAV8 Abs were measured in the donor plasma and cytokines were measured in the culture supernatants after 24 h of incubation with AAV8-null (Table 2). Transcriptomic changes were observed in major immune cell populations (Figure 5A), including CD4^+^ and CD8^+^ T cells, NK cells, and monocytes, following AAV8 treatment (Figure 5B). These changes were most pronounced after 24 h (Figure 5C). Analysis of individual donor data revealed that significant alterations (Figure 6) in immune cell populations on the UMAP after 24 h of AAV8 treatment were primarily observed in cells from two donors (D1 and D4) out of the six donors analyzed (Figure 5D). Several cell types contributed to the cytokine response to AAV8 (Figure 5E). Myeloid leukocytes and in particular monocytes upregulated transcripts for CCL2 (MCP-1), CCL3 (MIP-1α), CCL4 (MIP-1β), CXCL8 (IL-8), CXCL10 (IP-10), IL-1β, and TNF-α, while NK and T cells upregulated CCL3, CCL4, IFN-γ and TNF-α. IL-6 transcripts were weakly upregulated mainly in monocytes (data not shown). Furthermore, a strong activation of IFN-ɑ response genes was observed in myeloid leukocytes, NK, T, and B cells. However, no upregulation in IFN-ɑ transcripts could be detected in this setting, suggesting either that they were under the limit of detection with this technology, or that the IFN-ɑ secreting cells were too scarce or lost during PBMC isolation.

**Table 2:** Human whole blood (*n* = 6) was treated with 5e11 vg/mL of AAV8-null (genome without transgene) for 24 h; anti-AAV8 titers were measured by ELISA

Number	Anti-AAV8 Ig ELISA		Cytokine release in supernatant (Quanterix)				
	IgG titer	IgM titer	IFN-γ	IL-2	TNF-α	IL-8	IL-6
D1	<1/21870	<1/21870	41.5	10.9	3.9	7.5	71.0
D2	1/810	1/7290	1.5	1.5	3.0	21.9	30.8
D3	>1/10 (neg)	>1/10 (neg)	1	1	1.1	0.9	0.61
D4	<1/21870	1/30	35.2	8.2	1	4.1	27.7
D5	>1/10 (neg)	1/2430	1	1	1	1.1	1
D6	>1/10 (neg)	>1/10 (neg)	0.9	1	0.9	1.9	1

IFN-γ, IL-2 TNF-α, IL-8, and IL-6 were measured in the supernatants; the results are indicated in fold increase over PBS control.

Two of the six donors showed a strong increase in transcripts for IFN-γ and other cytokines 24 h after stimulation with rAAV8 (Figures 5C, 5D, and 5E). These donors displayed high titers of anti-AAV8 IgG (Table 2). They also showed a particular transcriptomic profile, with high percentages of CD4^+^ and CD8^+^ central memory T cells among leukocytes detected 24 h after AAV8 treatment (Figure 6A), suggestive of memory T cell responses. Indeed, differential gene expression at 4 and 24 h in CD4^+^ and CD8^+^ T cells was much stronger for these two donors than for the four other donors (Figures 6B and 6C). Four hours after treatment, while no significant change was observed in CD4^+^ T cells, CD8^+^ T cells displayed the stronger differential expression of genes involved in the nuclear factor (NF)-κB pathway (*RELB, BIRC3, NFKB1A*, and *NFKB2*) and in the immune response (*IL-27RA, POU2F2,* and *IFIT1*). After 24 h, both CD4^+^ and CD8^+^ T cells showed a highly significant increase in the expression of genes induced by IFN type I (*IFI6, ISG15, MX1, MX2, HERC5, TRIM22, IFI16, IFIT1,* and *OAS2*) and of other genes involved in the immune response (*STAT1, XAF1, RNF213,* and *SAMD9*). Important changes in gene expression were also observed in NK cells after 4 h (*NFKB1A, NFKB2, CCL4, POU2F2, MX1*, and *RELB*) and after 24 h (*ISG15, IFI6, MX1, MX2, HERC5, EIF2AK2, IFIT2, STAT1, IFIT3,* and *TRIM22)*. Monocytes also strongly induced the expression of genes involved in the immune response after 4 h (*CXCL3, CXCL2, SERPINB2, AHR, CLEC5A,* and *CXCL1*) and after 24 h (*MX1, ISG15, HERC5, SAMD9L, IFI6, DDX58, IFIT35, OAS1, OAS2,* and *IFIT1*) of AAV8-treatment (Table S1). Donor D2 had a relatively low titer of anti-AAV8 IgG and showed neither strong IFN-γ release, nor significant changes in transcriptomic profile.

## Discussion

Complement-mediated SAEs following systemic administration of rAAV have been documented in clinical trials.^24^^,^^30^^,^^33^^,^^34^ Prior research has indicated that rAAV vectors can activate the complement system, particularly in the presence of IgG.^27^^,^^29^ Our study corroborated that complement activation by rAAV2 and 8 was primarily dependent on anti-AAV Abs. It is noteworthy that not all the blood samples from seropositive individuals exhibited complement activation, suggesting that the specific type of immunoglobulin and/or the antibody-to-AAV particle ratio could influence this response. Nevertheless, we observed elevations in C3-des-Arg levels in a subset of donors who were seronegative for anti-AAV IgG and IgM. This may be attributable to the effect of other immunoglobulin subtypes, or rather to the activation of the alternative complement pathway. Results from the complement deposition assay through the alternative pathway confirmed the latter hypothesis. Indeed, previous studies have demonstrated that C3 complement components can bind directly to the AAV capsid, even in the absence of Abs.^35^ Products of the alternative pathway were also detected in non-human primates (NHPs) following systemic administration of AAV as well as in clinical trials, as reported by Salabarria et al.^26^ Clinically, an increase in complement activation via the alternative pathway has been described in two children who received onasemnogene abeparvovec and subsequently developed early symptoms of TMA.^24^ A recent study on patients treated with Zolgensma has shown that TMA in the context of AAV gene therapy is dependent on Abs (classical pathway) and is exacerbated by the alternative complement pathway.^16^^,^^26^^,^^36^^,^^37^

The potential for activation of the alternative complement pathway by rAAV may also be influenced by genetic variability. It is now recognized that variations in genes encoding complement components and regulatory proteins can affect the threshold for complement activation, rendering some patients more prone to alternative pathway activation.^30^^,^^38^ Importantly, complement activation can contribute to the initiation of adaptive immunity. Indeed Smith et al. showed that C3 inhibition with APL-9 lowers the innate immune response to the vector, with consequences on adaptive responses.^27^

Our study did not show any impact of the viral genome on complement activation. This finding implies that empty capsids, which are often present in AAV preparations, can activate the complement system just as effectively as full capsids, thereby increasing the risk of complement-mediated toxicities.

In our whole blood assay system, modest elevations in the release of a spectrum of cytokines, chemokines, and interferons were observed following stimulation with rAAV. The cytokines detected in response to rAAV2 included IP-10, MCP-1, MIP-1α, MIP-1β, IFN-γ, IL-2, IL-6, TNF-α, and IFN-α. Increases in several of these cytokines have also been reported following whole blood stimulation by other AAV serotypes.^22^^,^^27^ In response to rAAV8, the response was weaker and only significant for IP-10, MCP-1, MIP-1α, MIP-1β, IFN-γ, and IFN-α. Despite the higher dose of rAAV8 used in our whole blood assay, we observed fewer changes in cytokine, chemokine, and interferon profiles compared with rAAV2. This difference may be due to the distinct capsid structures of the two serotypes. Vandenberghe et al.^39^ have previously shown that AAV2 binds to DCs more efficiently than AAV8 due to its heparin binding site, also impacting the uptake by other cell types. Other factors may play a role, including the relatively lower titers of anti-AAV8 IgG compared with anti-AAV2 IgG within our study cohort as well as the ratios of capsid particles to IgG.

In mice, administration of a high rAAV8 dose led to increases in the levels of KC/GRO, MCP-1, MIP-1β, and IP-10. Despite some similarities, there are known differences between species in terms of immune response to rAAV.^40^ In human whole blood, we noted considerable inter-donor variability in cytokine and chemokine release upon incubation with rAAV. This heterogeneity is likely attributable to differences in pre-existing anti-capsid Ab levels, immunoglobulin subclasses,^40^^,^^41^ and other genetic factors, such as polymorphisms in Fc gamma receptors.^42^

We showed that the presence of anti-AAV Abs amplifies cytokine release in response to rAAV. Antibodies facilitate the uptake of the virus by immune cells, as previously demonstrated by Smith et al.^27^ Additionally, this enhancement may be consequent to the binding of immune complexes by Fc gamma receptors (FcɣR). The crosstalk between FcγRIIa and TLRs, as occurs upon recognition of IgG opsonized bacteria, has been shown to strongly amplify the production of pro-inflammatory cytokines.^43^

Our findings indicate that empty AAV capsids elicit less cytokine release than genome-containing AAV. For IFN-α, we observed increases with both AAV2-full and AAV8-full when compared with the corresponding empty capsids (Figures 3A and 3B) that were highly donor-dependent and did not reach statistical significance. However, for AAV2.7m8 (Figure 3C), statistically significant differences were observed between each genome-containing (full) capsid and the corresponding empty capsid, as well as between high-CpG (≥182 CpG) and low-CpG (135 CpG) genomes. The two experiments utilized three distinct AAV serotypes (AAV2, AAV8, and AAV2.7m8) that originated from distinct production batches, and that originated from separate production batches manufactured in different facilities. We hypothesize that the observed differences across serotypes may be attributed to variations in DNA contamination, though we were unable to confirm this due to the unavailability of data.

The strong correlation observed between IFN-α release and the CpG content of the genome supports the finding that Toll-like receptor 9 (TLR9) is central in this process.^20^^,^^21^^,^^22^^,^^34^^,^^44^ This underscores the critical importance of minimizing CpG motifs in the rAAV genome where possible to mitigate immune activation.^45^^,^^46^ Despite a strong induction of IFNɑ-response genes, we were not able to detect an upregulation of IFN-ɑ transcripts. This may be because plasmacytoid DCs, shown to sense unmethylated CpG motifs and secrete type I interferons in response to rAAV,^20^^,^^34^^,^^47^ are scarce in peripheral blood (0.2%–0.8% of total mononuclear cells) and therefore difficult to analyze with our employed transcriptomics methodology.

The study of immune cell populations involved in the cytokine and chemokine response to rAAV, dissected by scRNA-seq, highlights the contributions of monocytes, NK cells, T cells, and B cells. Our findings are in line with those of Kuranda et al., highlighting the role of NK cells and monocyte-derived dendritic cells (moDCs) in the immune response to rAAV. moDCs have been identified as the main cells secreting IL-6 and IL-1β in response to AAV in human blood.^19^ In our blood donor cohort, IL-6 release was significantly upregulated upon AAV2 treatment, while only few donor samples showed IL-6 release upon AAV8 treatment. Still, IL-6 and IL-1β gene expression were detected upon AAV8-treatment in monocytes and conventional dendritic cells.

Among the six donors analyzed by scRNA-seq, we saw two distinct response profiles. The two donors who had the highest titers of anti-AAV8 IgG showed strong and rapid IFN-γ responses. The proportion of CD4^+^ and CD8^+^ memory cells increased after 24 h of incubation with rAAV8, likely reflecting a memory T cell response.^48^ The other donors, who showed no increase in memory T cells, had lower titers or no anti-AAV8 Abs and showed less changes in their transcriptomic profiles. This again underscores the contribution of immune complexes to rAAV-dependent cytokine and chemokine release. Some of the donors with lower anti-AAV8 Ab titers did not show strong IFN-ɣ responses nor increases in memory T cell numbers upon rAAV incubation. Several studies already evidenced the absence of correlation between pre-existing cellular and humoral immune responses to rAAV.^11^^,^^47^^,^^48^^,^^49^

Our whole blood assay system was designed to model the peripheral immune response. However, it does not fully reflect the immune response occurring in specific tissues and organs, with varying resident immune cell populations as well as non-immune cell types, such as endothelial cells that are known to display complement regulators.^50^ Additional research using complex human organ models would be needed to have a more complete picture.

In summary, we have dissected the innate immune response to rAAV, characterized by complement activation and the release of cytokines by multiple cell types. Considering the pre-existing immune status of patients and carefully designing the vector genome is required to enhance the safety and efficacy of rAAV-mediated gene therapy. In current clinical trials, immunosuppressive agents such as corticosteroids, sirolimus, tacrolimus, mycophenolate mofetil, or cyclosporine are frequently employed, either prophylactically or reactively, with a primary focus on modulating T cell function.^51^ Progress in the knowledge of rAAV immunology will lead to the development of more targeted strategies to mitigate potential adverse effects while preserving therapeutic effectiveness.

## Materials and methods

### rAAV vectors

For the whole blood assays, rAAV2 and 8 (empty and null vectors) were produced in HEK293 cells produced by Vector Biolabs (Malvern, PA). AAV-null contains a genome with Inverted Terminal Repeats from AAV2, CMV promoter, and BGH polyA signal. For the *in vivo* experiment, AAV8-CAG-transgene (undisclosed) was produced by Virovek (Houston, TX) in HEK293 cells and purified through two rounds of CsCl ultra centrifugations. The CsCl was removed through buffer exchange with two PD-10 desalting columns. QC testing included purity check by SDS-PAGE, genomic titer by qPCR, anti-capsid ELISA, endotoxin test, and cryoelectron microscopy (except for AAV8-CAG-transgene). Endotoxin levels in the AAV batches were below 5 EU/mL for AAV2- and AAV8-empty and -null and below 0.06 EU/mL for AAV8-CAG-transgene.

rAAV2.7m8 empty capsids and containing different genomes were produced in HEK293 cells by Sirion. QC testing included purity check by SDS-PAGE, genomic titer by qPCR, anti-capsid ELISA, and endotoxin test.

For the anti-AAV Ab ELISA (see below), AAV2 and AAV8 empty capsids were produced by Virovek (Houston, TX) in insect cells, purified through two rounds of CsCl ultra centrifugations, and titrated by ELISA.

### Whole blood assay

Studies with human material from anonymized healthy donors were performed in accordance with the Declaration of Helsinki (Seventh Revision, 2013). Fresh whole blood from healthy human donors was collected in hirudin-coated (for complement activation measurement) and heparin-coated (for cytokine and chemokine measurement) blood collection tubes. To detect anti-AAV Abs by ELISA (see below), whole blood was spun down, and serum was harvested and stored at −80°C until use. Undiluted whole blood was distributed into 96-well plates and rAAV2, rAAV8 (empty or null), or rAAV2.7m8 was added to a final concentration of 1e10 viral particles (vp)/mL or 3e11 vp/mL (rAAV2) and 5e11 vp/mL (rAAV8 and rAAV2.7m8). Where indicated, intravenous immunoglobulins (IVIgs, Privigen, CSL Behring GmbH) were added at a final dilution of 1:100. As positive controls, blood was incubated with LPS (0.1 μg/mL, Invivogen), ODN2216 (10 μM, Invivogen), or zymosan (250 μg/mL, Sigma-Aldrich). Negative control wells were treated with the same PBS volume as the AAV suspension added to the treatment wells. Blood was incubated at 37°C in a 5% CO_2_ humidity incubator. All conditions were tested in duplicate (complement activation) or triplicate (cytokine and chemokine release). Supernatants were carefully collected from whole blood after 45 min (for complement activation measurements) or 24 h (for cytokine and chemokine measurements) post-treatment by centrifugation at 1,000 × *g* at 4°C. Samples for complement activation were diluted to 50% (v/v) in specimen stabilizing solution (A9576, QuidelOrtho). All samples were stored at −80°C until use.

### Human anti-AAV IgG/IgM ELISA

Detection of anti-AAV total IgG or IgM Abs in the plasma of healthy human donors was conducted using ELISA. Nunc Maxisorp P96 plates (Sigma-Aldrich) were coated overnight at 4°C with AAV2 or AAV8 empty capsids (Virovek) at 5e10 part/mL. After washing and plate saturation with blocking buffer (phosphate buffered saline [PBS, Gibco] + 2% [w/v] bovine serum albumin [Thermo Fisher]), seven-step 3-fold serial dilutions of plasma ranging from 10% (v/v) to 0.0046% (v/v) in PBS +1% (v/v) fetal calf serum (Serana) were incubated in the plates for 2 h at room temperature (RT). AP AffiniPure Goat Anti-Human IgG (Alkaline Phosphatase AffiniPure Goat Anti-Human IgG, Fcγ fragment specific, Jackson IR) or IgM (Alkaline Phosphatase AffiniPure Goat Anti-Human IgM, Fc5μ fragment specific, Jackson IR) were incubated for 1 h at RT. The signal was developed by adding *p*-nitrophenyl phosphate (Sigma) for 10 min at RT. Optical density (OD) was measured at 405 nm and 620 nm on a Cytation ELISA reader (BioTek). The positivity threshold was determined from the control pool of seronegative plasma by the mean optical density for each dilution +3 SD. Titers were determined as the last serum dilution above the positivity threshold. For IgG1 and IgG3 isotype detection, ELISA were performed as previously described.^50^

### Complement activation assay

Complement proteins C3a-des-Arg and C5a-des-Arg were measured by ELISA (Quidel A032 and A025, respectively) in accordance with the manufacturer’s protocols. Briefly, supernatants from whole blood assays were thawed and diluted (0.05% and 2.5%, v/v, respectively), added to the microassay plate, and incubated at RT for 1 h followed by three washing steps. Conjugates were added to the wells, incubated at RT for 1 h and washed three times. Next, substrate detection reagent was added to each well for 15 min at RT followed by stop solution. Optical densities were immediately read at 450 nm.

### Complement deposition assay

To assess whether AAV2-induced complement activation in donor sera was dependent on a specific pathway, complement deposition assays on AAV2 capsids were performed, adapted from De Boer et al. (2023)^31^ with appropriate buffers containing either the Ca^2+^ or Mg2^+^ ions necessary for the activation of the classical or alternative pathways, respectively.^52^ All incubation steps were performed with a volume of 100 μL, at RT, while shaking for 1 h. Each wash step comprised five times automated washing with 0.02% (v/v) Tween 20 in PBS on an Elx405 Plate washer (BioTek Instruments).

For measuring complement activation through the classical pathway, AAV2 empty was coated at 5e10 vp/mL as described above. As positive control, wells were coated with 3 μg/mL human IgM (Sigma-Aldrich). After washing, plates were incubated with a 1.5-fold dilution series of sera of individual healthy donors, starting at 10% (v/v) serum diluted in Veronal buffer 0.1% (v/v) Tween-20, 0.3% (w/v) BSA, 1 mM CaCl_2_, and 0.5 mM MgCl_2_. For each serum sample, a negative control was included, consisting of 10% (v/v) serum in Veronal buffer on uncoated wells. After incubation, supernatants were collected for fluid phase C3a-des-Arg and C5a-des-Arg measurement as described above and plates were washed. Next, 0.55 μg/mL biotinylated anti-C3.19 (Sanquin Research) in high performance ELISA buffer (HPE, Essange Reagents) was added. After incubation and washing, plates were incubated with 0.01% (v/v) streptavidin-horseradish peroxidase (Cytiva), diluted in HPE, for 20 min. After washing, the plates were developed using 1-step Ultra TMB-ELISA (Thermo Fisher Scientific) according to manufacturer’s instructions, stopping the reaction with 0.2 M H_2_SO_4_. Absorbance at 450 nm was measured on a Synergy 2 Multi-Mode Plate reader (BioTek Instruments) and corrected for background absorbance at 540 nm.

For measuring complement activation through the alternative pathway, AAV2 empty was coated at 5e10 vp/mL in CB on Nunc Polysorp 96-wells plates as described above. As positive controls, wells were coated with 5 μg/mL *Salmonella typhosa* LPS (Sigma-Aldrich). Sera were serially diluted using a 1.5-fold dilution steps starting at 25% (v/v), in Veronal buffer containing 0.1% (v/v) Tween-20, 0.3% (w/v) BSA, 5 mm MgCl_2_, and 10 mm EDTA. All other steps were performed as described above for the classical complement activation assay.

### Cytokine, chemokine, and interferon assessment

Pro-inflammatory cytokines levels were measured in the supernatants from whole blood assays using V-PLEX Pro-inflammatory Panel 1 Human Kit (Mesoscale Diagnostics, K15049D), chemokine levels were measured using Chemokine Panel 1 Human Kit (Mesoscale Diagnostics, K15047D) and interferon levels were measured using U-PLEX Interferon Combo(hu) (Mesoscale Diagnostics, K15094K). Assays were performed according to the manufacturer’s recommendations. Cytokine and chemokine concentrations were determined from the calibration control standard curve using the Meso Scale Discovery Workbench 4.0 software. After three washes of the plates, the diluted samples (50%, v/v in dilution buffer) and calibrator controls were added to the plates. After 2 h incubation at RT while shaking at 550 rpm, the plates were washed three times. The SULFO-TAG-conjugated detection Abs were then incubated in the plates for 1 h at RT while shaking. After three washes, reading buffer was added and the plates were immediately read on Meso QuickPlex SQ 120MM (Mesoscale Diagnostics) and analyzed as described above.

For the comparison of supernatant cytokine concentrations across various conditions (Figures 3A and 3B), linear mixed effects models (LME) were fitted separately for each cytokine, using the log transformed cytokine concentration as target variable and including all factor interactions that can be calculated given the specific experiment design (fixed effect part AAV type∗ Capsid ∗ IVIg + AAV Concentration ∗ Capsid ∗ IVIg) and a random intercept for every cell donor in the model formula. Furthermore, heteroscedasticity across groups was taken into account by allowing different residual variance for each group. Contrasts of interest, i.e., IVIg+ vs. IVIg− for each combination of AAV, AAV concentration, and capsid were calculated, adjusting *p* values globally for multiplicity of testing using the mvt (multivariate t-distribution) method. For the comparison of IFN-α concentrations across CpG motifs (Figure 3C), the log scaled concentration was fitted with a simpler LME using the group indicator as a fixed effect together with a random intercept by cell donor. Variance heterogeneity in this case was described by an exponential variance function structure, i.e., describing group variability as a function of the fitted group means. All pairwise comparisons across groups were performed and *p* values adjusted by the Tukey method. All analyses were carried out in R version 4.3.0 [×1], using packages nlme [×2] and emmeans [×3]. [×1] R Core Team (2023). R: A Language and Environment for Statistical Computing. R Foundation for Statistical Computing, Vienna, Austria. <https://www.R-project.org/> [×2] Pinheiro J, Bates D, R Core Team (2025). nlme: Linear and Nonlinear Mixed Effects Models. R package version 3.1–167, <https://CRAN.R-project.org/package=nlme> [×3] Lenth R (2024). emmeans: Estimated Marginal Means, aka Least-Squares Means. R package version 1.10.6, <https://CRAN.R-project.org/package=emmeans>.

### Single-cell transcriptome library preparation

Fresh whole blood from healthy human donors was collected in heparin lithium-coated blood collection tubes. Blood was plated in a 6-well plate and rAAV8 was added at a final concentration of 5e11 viral genome (vg)/mL. After incubation for 4 h or 24 h at 37°C in a 5% CO_2_ incubator, supernatants were harvested. Cytokine levels (IFN-γ, IL-8, TNF-ɑ, IL-2, and IL-6) in the supernatants were measured with the Human 5-plex array (Quanterix). PBMCs isolated from whole blood samples using EasySep Direct human PBMC isolation kit (Stemcell) were assessed for cell number and viability. For each sample, suspensions of 800 cells/μL were prepared as input for GEM (Gel beads in Emulsion) generation with a target recovery of 8,000 cells. GEM generation and library preparation were performed on the Chromium Controller (10× Genomics, Aventura, FL) using Single Cell 3′ dual index reagent kit v3.1 (10× Genomics) according to the manufacturer’s instructions. Briefly, after GEM generation, a post GEM-reverse transcriptase cleanup and cDNA amplification was performed. The quality control and quantification of cDNA were assessed using a high sensitivity Bioanalyzer Chip (Agilent Technologies, Santa Clara, CA) before sample indexing using the Dual Index Kit TT Set A (10× Genomics). The final libraries were assessed for quality and concentration using a Bioanalyzer HS Assay kit (Agilent Technologies) and Qubit dsDNA HS assay kit (Thermo Fisher, Waltham, MA).

### Short read sequencing

A 2.5-nM equimolar pool of 10 × 3′ scRNA-seq libraries was prepared prior to paired-end sequencing on a Novaseq 6000 (Illumina, San Diego, CA) using a 100-cycle paired-end kit and S2 flow cell (Illumina). A read depth of 50 million reads per sample was targeted. The 10 × 3′ single-cell library constructs use standard Illumina P5 and P7 adaptors. The 16-base pair (bp) 10× barcodes and 12-bp unique molecular index (UMI) are both sequenced in Read 1. The eight-bp sample index sequences incorporated as the sample index read 90 bp of the insert are sequenced in Read 2. TruSeq Read 1 and Read 2 are standard Illumina sequencing primer sites used in paired-end sequencing. Sequencing the libraries produces a standard Illumina BCL data output folder. Demultiplexing and fastq files are created using the CellRanger 6.0.2 mkfastq+count workflow v1.1 in Arvados.

### Sequencing analysis

Fastq files were aligned to the human transcriptome (GRCh38) using CellRanger count v6.0.2 with the parameters ‘--expect-cells = 6000’. All cells showing >200 counts were further merged across all samples and processed with scanpy and the besca standard workflow.^53^ Filtering was performed with the parameters min_genes = 600, min_cells = 20, min_counts = 1000, n_genes = 6000, percent_mito = 0.15, max_counts = 40000. In brief, RNA counts were normalized per 10,000, the topmost highly variable genes were selected, total gene and mitochondrial reads were regressed out, PCA was performed, and the first 50 principal components were used for nearest-neighbor calculations and Leiden clustering, as well as for UMAP-based visualization. For annotating the samples, a first version of the analysis was run using bbknn^54^ for integration (with time as batch). Annotation was performed using besca’s sig-annot module and annotations transferred to a second version of the analysis with no integration/batch correction, which was used for all downstream analyses. Differential expression analysis was performed per cell type (at distinct annotation levels) on pseudobulk expression profiles using limma voom^55^ 3.54.2 in R 4.2.2 and the model ∼0 + treatment + donor, both across all samples and separately for donors showing an IFN-ɣ response (D1 and D4) and the rest (D2, D3, D5, and D6). Jupyter notebooks and R markdowns are available for data preprocessing, clustering and visualization, cell annotation, and differential expression. The data are available in ArrayExpress https://www.ebi.ac.uk/biostudies/arrayexpress/studies/E-MTAB-14760. The detailed analysis is available online: https://github.com/bedapub/AAVimmuneresponse_scRNAseq_PBMC_publication.

### *In vivo* study

The animal study design is in general compliance with the permission of the Swiss Cantonal Veterinary Office. The test facility is Association for Assessment and Accreditation of Laboratory Animal Care accredited. Male C57BL/6J mice were dosed intravenously into the tail vein with an AAV8 vector driving transgene expression under the control of a CAG promoter, at a dose of 4.04e14 vg/kg or vehicle (50mM Tris, 150 mM NaCl, 1% Sorbitol, 0.5% Sucrose, 0.001% F-68, pH 7.4). Blood (100 μL) was sampled through the tail vein 1 day before AAV dosing, 2 h, 24 h, and 14 days after dosing and processed to serum. Cytokine and chemokine were determined using a U-PLEX custom murine panel with an MESO QuickPlex SQ 120 instrument (Meso Scale Discovery). The calculated fold increase corresponds to the ratio of the signal for the given time point on the signal obtained at day 1. Significance was determined by processing the complete cyto/chemokine dataset in a single generalized linear model of the form: cyto_model = gls(log10(Signal) ∼ Cond∗Cytokine, corr = corAR1(form = ∼1| AnimalID), weights = varIdent(form = ∼1|Cytokine∗Treatment),data = tmp, method = “REML”) where a Cond(ition) is defined by a specific combination of the treatment and time point of observation. Multiple testing adjustment (final correction of *p* values) is done with the “mvt” method, standing for “multivariate t-distribution” method.

## Data availability

The data that support the key findings of this study are available within the article and supplemental material or upon request from the corresponding author, Rebecca Xicluna (rebecca.xicluna@roche.com).

## Acknowledgments

This work was funded by Roche Pharma R&D. The graphical abstract was done on BioRender.

## Author contributions

J.J.F.-v.H., A.W.T., R.B.P., T.S., E.B., R.X., and H.H. designed the experiments; J.J.F.-v.H., T.R., D.V.T., M.C.B., R.X., and E.S. conducted the experiments; C.B.-L., H.H., M.B.O., and R.X. designed the *in vivo* study; G.S. and P.C.S. did the scRNA-seq and statistical analysis; R.X. and H.H. wrote the manuscript.

## Declaration of interests

T.S., E.B., R.X., H.H., D.V.T., E.S., C.B.-L., M.B.O., G.S., and P.C.S. were employed by Roche. The authors were employed by Sanquin Diagnostic Services.

## Supplemental information

Document S1. Figures S1–S4 and Table S1

Document S2. Article plus supplemental information

## References

[1] Hoy S.M. (2019). Onasemnogene Abeparvovec: First Global Approval. Drugs.

[2] Blair H.A. (2022). Valoctocogene Roxaparvovec: First Approval. Drugs.

[3] Commissioner O. (2023). FDA Approves First Gene Therapy for Treatment of Certain Patients with Duchenne Muscular Dystrophy.

[4] Blacklow N.R., Hoggan M.D., Kapikian A.Z., Austin J.B., Rowe W.P. (1968). Epidemiology of adenovirus-associated virus infection in a nursery population. Am. J. Epidemiol..

[5] Atchison R.W., Casto B.C., Hammon W.M. (1966). Electron microscopy of adenovirus-associated virus (AAV) in cell cultures. Virology.

[6] Vandamme C., Adjali O., Mingozzi F. (2017). Unraveling the Complex Story of Immune Responses to AAV Vectors Trial After Trial. Hum. Gene Ther..

[7] Mendell J.R., Connolly A.M., Lehman K.J., Griffin D.A., Khan S.Z., Dharia S.D., Quintana-Gallardo L., Rodino-Klapac L.R. (2022). Testing preexisting antibodies prior to AAV gene transfer therapy: rationale, lessons and future considerations. Mol. Ther. Methods Clin. Dev..

[8] Earley J., Piletska E., Ronzitti G., Piletsky S. (2023). Evading and overcoming AAV neutralization in gene therapy. Trends Biotechnol..

[9] Mingozzi F., Maus M.V., Hui D.J., Sabatino D.E., Murphy S.L., Rasko J.E.J., Ragni M.V., Manno C.S., Sommer J., Jiang H. (2007). CD8+ T-cell responses to adeno-associated virus capsid in humans. Nat. Med..

[10] Manno C.S., Pierce G.F., Arruda V.R., Glader B., Ragni M., Rasko J.J., Ozelo M.C., Hoots K., Blatt P., Konkle B. (2006). Successful transduction of liver in hemophilia by AAV-Factor IX and limitations imposed by the host immune response. Nat. Med..

[11] Vandamme C., Xicluna R., Hesnard L., Devaux M., Jaulin N., Guilbaud M., Le Duff J., Couzinié C., Moullier P., Saulquin X., Adjali O. (2019). Tetramer-Based Enrichment of Preexisting Anti-AAV8 CD8+ T Cells in Human Donors Allows the Detection of a TEMRA Subpopulation. Front. Immunol..

[12] Veron P., Leborgne C., Monteilhet V., Boutin S., Martin S., Moullier P., Masurier C. (2012). Humoral and cellular capsid-specific immune responses to adeno-associated virus type 1 in randomized healthy donors. J. Immunol..

[13] Zaiss A.-K., Liu Q., Bowen G.P., Wong N.C.W., Bartlett J.S., Muruve D.A. (2002). Differential activation of innate immune responses by adenovirus and adeno-associated virus vectors. J. Virol..

[14] Carestia A., Kim S.-J., Horling F., Rottensteiner H., Lubich C., Reipert B.M., Crowe B.A., Jenne C.N. (2021). Modulation of the liver immune microenvironment by the adeno-associated virus serotype 8 gene therapy vector. Mol. Ther. Methods Clin. Dev..

[15] Hinderer C., Katz N., Buza E.L., Dyer C., Goode T., Bell P., Richman L.K., Wilson J.M. (2018). Severe Toxicity in Nonhuman Primates and Piglets Following High-Dose Intravenous Administration of an Adeno-Associated Virus Vector Expressing Human SMN. Hum. Gene Ther..

[16] Hordeaux J., Lamontagne R.J., Song C., Buchlis G., Dyer C., Buza E.L., Ramezani A., Wielechowski E., Greig J.A., Chichester J.A. (2024). High-dose systemic adeno-associated virus vector administration causes liver and sinusoidal endothelial cell injury. Mol. Ther..

[17] Flotte T.R. (2020). Revisiting the “New” Inflammatory Toxicities of Adeno-Associated Virus Vectors. Hum. Gene Ther..

[18] Lek A., Wong B., Keeler A., Blackwood M., Ma K., Huang S., Sylvia K., Batista A.R., Artinian R., Kokoski D. (2023). Death after High-Dose rAAV9 Gene Therapy in a Patient with Duchenne’s Muscular Dystrophy. N. Engl. J. Med..

[19] Kuranda K., Jean-Alphonse P., Leborgne C., Hardet R., Collaud F., Marmier S., Costa Verdera H., Ronzitti G., Veron P., Mingozzi F. (2018). Exposure to wild-type AAV drives distinct capsid immunity profiles in humans. J. Clin. Investig..

[20] Rogers G.L., Shirley J.L., Zolotukhin I., Kumar S.R.P., Sherman A., Perrin G.Q., Hoffman B.E., Srivastava A., Basner-Tschakarjan E., Wallet M.A. (2017). Plasmacytoid and conventional dendritic cells cooperate in crosspriming AAV capsid-specific CD8+ T cells. Blood.

[21] Ashley S.N., Somanathan S., Giles A.R., Wilson J.M. (2019). TLR9 signaling mediates adaptive immunity following systemic AAV gene therapy. Cell. Immunol..

[22] Alakhras N.S., Moreland C.A., Wong L.C., Raut P., Kamalakaran S., Wen Y., Siegel R.W., Malherbe L.P. (2024). Essential role of pre-existing humoral immunity in TLR9-mediated type I IFN response to recombinant AAV vectors in human whole blood. Front. Immunol..

[23] Hösel M., Broxtermann M., Janicki H., Esser K., Arzberger S., Hartmann P., Gillen S., Kleeff J., Stabenow D., Odenthal M. (2012). Toll-like receptor 2-mediated innate immune response in human nonparenchymal liver cells toward adeno-associated viral vectors. Hepatol. Baltim. Md.

[24] Chand D.H., Zaidman C., Arya K., Millner R., Farrar M.A., Mackie F.E., Goedeker N.L., Dharnidharka V.R., Dandamudi R., Reyna S.P. (2021). Thrombotic Microangiopathy Following Onasemnogene Abeparvovec for Spinal Muscular Atrophy: A Case Series. J. Pediatr..

[25] Mendell J.R., Al-Zaidy S.A., Rodino-Klapac L.R., Goodspeed K., Gray S.J., Kay C.N., Boye S.L., Boye S.E., George L.A., Salabarria S. (2021). Current Clinical Applications of In Vivo Gene Therapy with AAVs. Mol. Ther..

[26] Salabarria S.M., Corti M., Coleman K.E., Wichman M.B., Berthy J.A., D’Souza P., Tifft C.J., Herzog R.W., Elder M.E., Shoemaker L.R. (2024). Thrombotic microangiopathy following systemic AAV administration is dependent on anti-capsid antibodies. J. Clin. Investig..

[27] Smith C.J., Ross N., Kamal A., Kim K.Y., Kropf E., Deschatelets P., Francois C., Quinn W.J., Singh I., Majowicz A. (2022). Pre-existing humoral immunity and complement pathway contribute to immunogenicity of adeno-associated virus (AAV) vector in human blood. Front. Immunol..

[28] Pouw R.B., Ricklin D. (2021). Tipping the balance: intricate roles of the complement system in disease and therapy. Semin. Immunopathol..

[29] West C., Federspiel J.D., Rogers K., Khatri A., Rao-Dayton S., Ocana M.F., Lim S., D’Antona A.M., Casinghino S., Somanathan S. (2023). Complement Activation by Adeno-Associated Virus-Neutralizing Antibody Complexes. Hum. Gene Ther..

[30] Kropf E., Markusic D.M., Majowicz A., Mingozzi F., Kuranda K. (2024). Complement system response to adeno-associated virus (AAV) vector gene therapy. Hum. Gene Ther..

[31] de Boer E.C., Thielen A.J., Langereis J.D., Kamp A., Brouwer M.C., Oskam N., Jongsma M.L., Baral A.J., Spaapen R.M., Zeerleder S. (2023). The contribution of the alternative pathway in complement activation on cell surfaces depends on the strength of classical pathway initiation. Clin. Transl. Immunology.

[32] Vidarsson G., Dekkers G., Rispens T. (2014). IgG subclasses and allotypes: from structure to effector functions. Front. Immunol..

[33] Guillou J., de Pellegars A., Porcheret F., Frémeaux-Bacchi V., Allain-Launay E., Debord C., Denis M., Péréon Y., Barnérias C., Desguerre I. (2022). Fatal thrombotic microangiopathy case following adeno-associated viral SMN gene therapy. Blood Adv..

[34] Cao D., Byrne B.J., de Jong Y.P., Terhorst C., Duan D., Herzog R.W., Kumar S.R.P. (2024). Innate Immune Sensing of Adeno-Associated Virus Vectors. Hum. Gene Ther..

[35] Zaiss A.K., Cotter M.J., White L.R., Clark S.A., Wong N.C.W., Holers V.M., Bartlett J.S., Muruve D.A. (2008). Complement Is an Essential Component of the Immune Response to Adeno-Associated Virus Vectors. J. Virol..

[36] Palazzi X., Pardo I.D., Sirivelu M.P., Newman L., Kumpf S.W., Qian J., Franks T., Lopes S., Liu J., Monarski L. (2022). Biodistribution and Tolerability of AAV-PHP.B-CBh-SMN1 in Wistar Han Rats and Cynomolgus Macaques Reveal Different Toxicologic Profiles. Hum. Gene Ther..

[37] Hudry E., Aihara F., Meseck E., Mansfield K., McElroy C., Chand D., Tukov F.F., Penraat K. (2023). Liver injury in cynomolgus monkeys following intravenous and intrathecal scAAV9 gene therapy delivery. Mol. Ther..

[38] Rodríguez de Córdoba S. (2023). Genetic variability shapes the alternative pathway complement activity and predisposition to complement-related diseases. Immunol. Rev..

[39] Vandenberghe L.H., Wang L., Somanathan S., Zhi Y., Figueredo J., Calcedo R., Sanmiguel J., Desai R.A., Chen C.S., Johnston J. (2006). Heparin binding directs activation of T cells against adeno-associated virus serotype 2 capsid. Nat. Med..

[40] Murphy S.L., Li H., Mingozzi F., Sabatino D.E., Hui D.J., Edmonson S.A., High K.A. (2009). Diverse IgG subclass responses to adeno-associated virus infection and vector administration. J. Med. Virol..

[41] Fitzpatrick Z., Leborgne C., Barbon E., Masat E., Ronzitti G., van Wittenberghe L., Vignaud A., Collaud F., Charles S., Simon Sola M. (2018). Influence of Pre-existing Anti-capsid Neutralizing and Binding Antibodies on AAV Vector Transduction. Mol. Ther. Methods Clin. Dev..

[42] Hussain K., Hargreaves C.E., Rowley T.F., Sopp J.M., Latham K.V., Bhatta P., Sherington J., Cutler R.M., Humphreys D.P., Glennie M.J. (2019). Impact of Human FcγR Gene Polymorphisms on IgG-Triggered Cytokine Release: Critical Importance of Cell Assay Format. Front. Immunol..

[43] Vogelpoel L.T.C., Baeten D.L.P., de Jong E.C., den Dunnen J. (2015). Control of Cytokine Production by Human Fc Gamma Receptors: Implications for Pathogen Defense and Autoimmunity. Front. Immunol..

[44] Martino A.T., Suzuki M., Markusic D.M., Zolotukhin I., Ryals R.C., Moghimi B., Ertl H.C.J., Muruve D.A., Lee B., Herzog R.W. (2011). The genome of self-complementary adeno-associated viral vectors increases Toll-like receptor 9-dependent innate immune responses in the liver. Blood.

[45] Wright J.F. (2020). Quantification of CpG Motifs in rAAV Genomes: Avoiding the Toll. Mol. Ther..

[46] Wright J.F. (2020). Codon Modification and PAMPs in Clinical AAV Vectors: The Tortoise or the Hare?. Mol. Ther..

[47] Kruzik A., Fetahagic D., Hartlieb B., Dorn S., Koppensteiner H., Horling F.M., Scheiflinger F., Reipert B.M., de la Rosa M. (2019). Prevalence of Anti-Adeno-Associated Virus Immune Responses in International Cohorts of Healthy Donors. Mol. Ther. Methods Clin. Dev..

[48] Xicluna R., Avenel A., Vandamme C., Devaux M., Jaulin N., Couzinié C., Le Duff J., Charrier A., Guilbaud M., Adjali O., Gernoux G. (2024). Prevalence Study of Cellular Capsid-Specific Immune Responses to AAV2, 4, 5, 8, 9, and rh10 in Healthy Donors. Hum. Gene Ther..

[49] Pabinger I., Ayash-Rashkovsky M., Escobar M., Konkle B.A., Mingot-Castellano M.E., Mullins E.S., Negrier C., Pan L., Rajavel K., Yan B., Chapin J. (2024). Multicenter assessment and longitudinal study of the prevalence of antibodies and related adaptive immune responses to AAV in adult males with hemophilia. Gene Ther..

[50] Noris M., Galbusera M. (2023). The complement alternative pathway and hemostasis. Immunol. Rev..

[51] Chu W.S., Ng J. (2021). Immunomodulation in Administration of rAAV: Preclinical and Clinical Adjuvant Pharmacotherapies. Front. Immunol..

[52] Gupta P., Tripathy A.S. (2020). Alternative pathway of complement activation has a beneficial role against Chandipura virus infection. Med. Microbiol. Immunol..

[53] Steenhuis M., van Mierlo G., Derksen N.I., Ooijevaar-de Heer P., Kruithof S., Loeff F.L., Berkhout L.C., Linty F., Reusken C., Reimerink J. (2021). Dynamics of antibodies to SARS-CoV-2 in convalescent plasma donors. Clin. Transl. Immunology.

[54] Polański K., Young M.D., Miao Z., Meyer K.B., Teichmann S.A., Park J.-E. (2020). BBKNN: fast batch alignment of single cell transcriptomes. Bioinformatics.

[55] Law C.W., Chen Y., Shi W., Smyth G.K. (2014). voom: precision weights unlock linear model analysis tools for RNA-seq read counts. Genome Biol..

