# Supplementary material for: Innate immune response to AAV-based gene therapy vectors: Mechanisms of complement activation and cytokine release
Source: Mol Ther Methods Clin Dev. 2025 Aug 12;33(3):101551. doi: 10.1016/j.omtm.2025.101551 (PMC12409308; doi:10.1016/j.omtm.2025.101551)
Supplement: Document S1. Figures S1–S4 and Table S1 [file mmc1.pdf]

OMTM, Volume *33*

Supplemental information

Innate immune response to AAV-based gene therapy vectors: Mechanisms of complement activation and cytokine release

Rebecca Xicluna, Petra C. Schwalie, Emma Bell, Desiree Von Tell, Guido Steiner, Emily Seeger, Julian J. Freen-van Heeren, Annelies W. Turksma, Richard B. Pouw, Timo Schwandt, Michael B. Otteneder, Theo Rispens, Mieke C. Brouwer, Cristina Bertinetti-Lapatki, and Hélène Haegel

Cell type	CD4^{+} T cells		CD8^{+} T cells		CD8^{+} T cells		NK cells		NK cells		Monocytes		Monocytes	
Time after treatment	24h		4h		24h		4h		24h		4h		24h	
	Gene	p-value	Gene	p-value	Gene	p-value	Gene	p-value	Gene	p-value	Gene	p-value	Gene	p-value
Top genes upregulated upon AAV treatment	IFI6	2.00E-19	RELB	2.90E-11	ISG15	8.90E-29	BIRC3	4.50E-11	ISG15	1.20E-35	CXCL3	2.90E-09	MX1	6.70E-14
	IFI44L	4.80E-19	BIRC3	6.30E-11	IFI6	1.30E-27	NFKBIA	2.40E-10	IFI6	5.00E-32	SERPINB2	6.90E-09	ISG15	7.00E-14
	ISG15	7.10E-19	NFKB2	2.20E-09	MX1	5.00E-26	NFKB2	4.60E-10	MX1	2.00E-30	RGL1	1.00E-08	LY6E	5.30E-13
	MX2	3.30E-18	NFKBIA	2.90E-08	MX2	3.30E-25	IER5	1.40E-09	MX2	3.50E-29	CXCL2	2.60E-08	NT5C3A	8.20E-13
	LY6E	1.20E-17	FEZ1	3.50E-07	STAT1	5.70E-25	RELB	3.20E-09	HERC5	6.50E-28	SQLE	1.10E-07	SAMD9L	9.20E-13
	MX1	5.50E-17	IL27RA	3.50E-07	LY6E	1.00E-24	OTULIN	2.00E-08	EIF2AK2	1.00E-27	CLEC5A	1.40E-07	HERC5	1.00E-12
	XAF1	9.20E-17	POU2F2	3.60E-07	HERC5	1.30E-23	FEZ1	5.90E-08	IFIT2	4.10E-27	AHR	1.50E-07	DDX58	1.10E-12
	TRIM22	1.00E-16	IFIT1	4.80E-07	TRIM22	1.70E-23	CCL4	1.10E-07	LY6E	7.30E-27	CXCL1	1.50E-07	IFITM3	1.90E-12
	IFIT1	1.50E-16	NFKB1	8.00E-07	IFIT2	1.90E-23	LYST	2.80E-07	STAT1	9.10E-27	NOP58	1.80E-07	IFI6	2.10E-12
	HERC5	1.90E-16	BCL3	1.2E-06	IFIT1	2.20E-23	POU2F2	5.00E-07	PLSCR1	3.20E-26	MAFB	3.40E-07	MX2	3.30E-12
	PNPT1	2.10E-16	IER5	1.6E-06	XAF1	1.10E-22	MX1	5.90E-07	IFIT3	6.80E-26	TNFSF15	3.50E-07	TNFSF10	3.40E-12
	OAS2	2.60E-16	LTB	0.000013	OAS2	1.60E-22	NINJ1	1.2E-06	TRIM22	2.30E-25	ABCE1	3.70E-07	RSAD2	3.40E-12
	DDX58	5.10E-16	TRAF3	0.00012	BST2	1.90E-22	CCL4L2	4.6E-06	BST2	2.40E-25	SLC7A1	4.10E-07	IFIH1	3.80E-12
	RNF213	5.20E-16	MX1	0.00012	EIF2AK2	2.20E-22	ANP32A	7.6E-06	ISG20	2.90E-25	TRMT6	4.20E-07	SAMD9	3.80E-12
	MT2A	5.60E-16	NINJ1	0.00015	PLSCR1	2.30E-22	LACTB	0.000011	IFITM3	3.80E-25	NOLC1	5.00E-07	IFI35	4.40E-12
	STAT1	5.70E-16	ARRDC3	0.00015	PARP9	2.30E-22	CCL3	0.000013	SAMD9L	4.20E-25	HNRNPAB	6.20E-07	OAS2	8.00E-12
	EIF2AK2	6.70E-16	TNIP1	0.00015	SAMD9	3.50E-22	NFKB1	0.000014	SAMD9	9.80E-25	MARCKS	6.50E-07	OAS1	8.50E-12
	OAS1	8.20E-16	OPTN	0.00017	IFITM2	4.30E-22	IFNG	0.000015	XAF1	1.10E-24	CD163	7.10E-07	IFITM2	9.40E-12
	SAMD9	8.70E-16	RSAD2	0.00021	IFI35	4.50E-22	IFIT1	0.000018	EPSTI1	1.20E-24	PNO1	7.40E-07	XAF1	1.20E-11
	DDX60	1.10E-15	WAKMAR2	0.00022	MT2A	7.10E-22	RCC2	0.000023	OAS1	2.00E-24	EDN1	7.40E-07	IFIT1	1.70E-11
	IFI35	1.30E-15	CCL2	0.00023	IFI16	4.30E-21	ABCF1	0.000023	MT2A	2.90E-24	NLN	7.50E-07	TNFSF13B	3.40E-11
	DDX60L	1.30E-15	IFIT2	0.00029	PARP14	6.20E-21	TNFRSF9	0.000025	IFI35	4.60E-24	PLA2G7	8.20E-07	UBE2L6	4.30E-11
	EPSTI1	1.40E-15	IFIH1	0.00038	RNF213	6.50E-21	RPL17	0.000026	IRF7	7.00E-24	GPATCH4	8.50E-07	CMPK2	4.60E-11
	BST2	1.80E-15	STARD10	0.00039	OASL	7.20E-21	IL4R	0.000027	PARP14	9.20E-24	SLC7A11	9.80E-07	ISG20	4.80E-11
	IFI16	3.90E-15	MIDEAS	0.00045	DTX3L	1.10E-20	CCL2	0.00003	OAS2	2.80E-23	SLC4A7	0.000001	IRF7	5.50E-11

Table S1: Single cell RNA-sequencing was performed on human PBMCs isolated from whole blood and treated with 5e11 vg/mL of AAV8-null (genome without transgene). The top 25 genes upregulated in IFN-γ positive donors upon AAV8 treatment are shown for the indicated cell types at 4 hrs and 24 hrs, with the significance (p-value) for each upregulated gene.

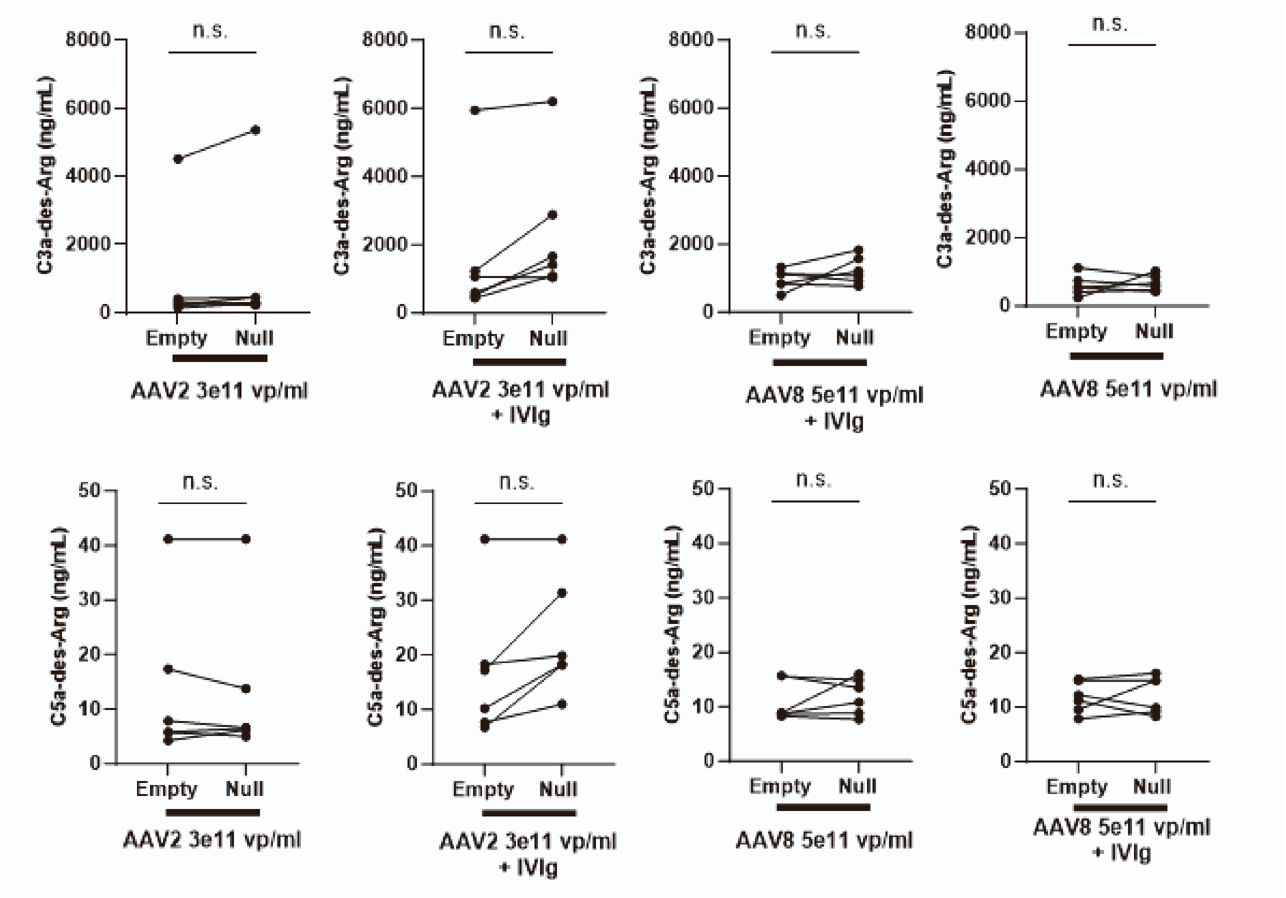

Figure S1:

Blood from healthy donors (n=6) was incubated with different concentrations of AAV2 or AAV8 empty capsids or null (CMV-no transgene-polyA) in presence or in absence of IVIg. After 45 min, C3a-des-arg and C5a-des-Arg concentration were measured in supernatants by ELISA. IVIg: Intravenous immune globulin. Significance was determined by the Wilcoxon test for paired samples. N.s. non significant

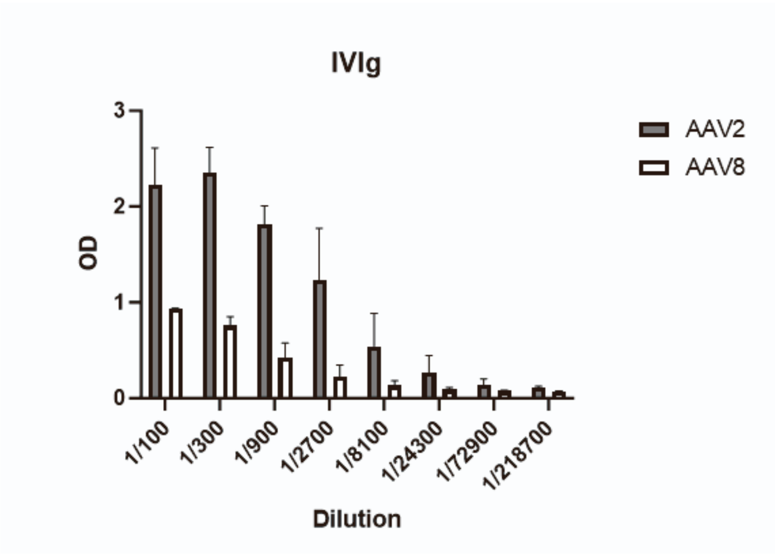

Figure S2: Anti-AAV2 and anti-AAV8 IgG levels in IVIg

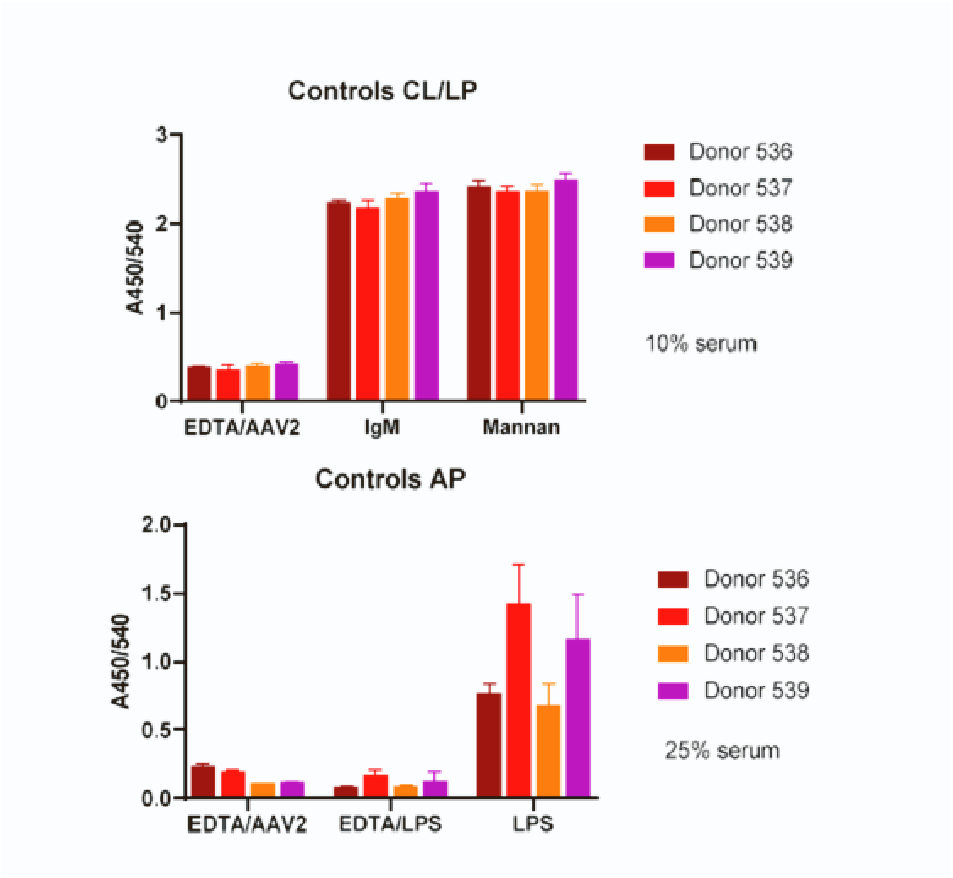

Figure S3: Control C3 deposition assay. Serum of four donors was tested for C3 deposition in buffers that allow activation of either the classical and lectin pathways, or the alternative pathway. Sera were tested with EDTA/AAV2 and EDTA LPS (negative control) , IgM, Mannan and LPS.

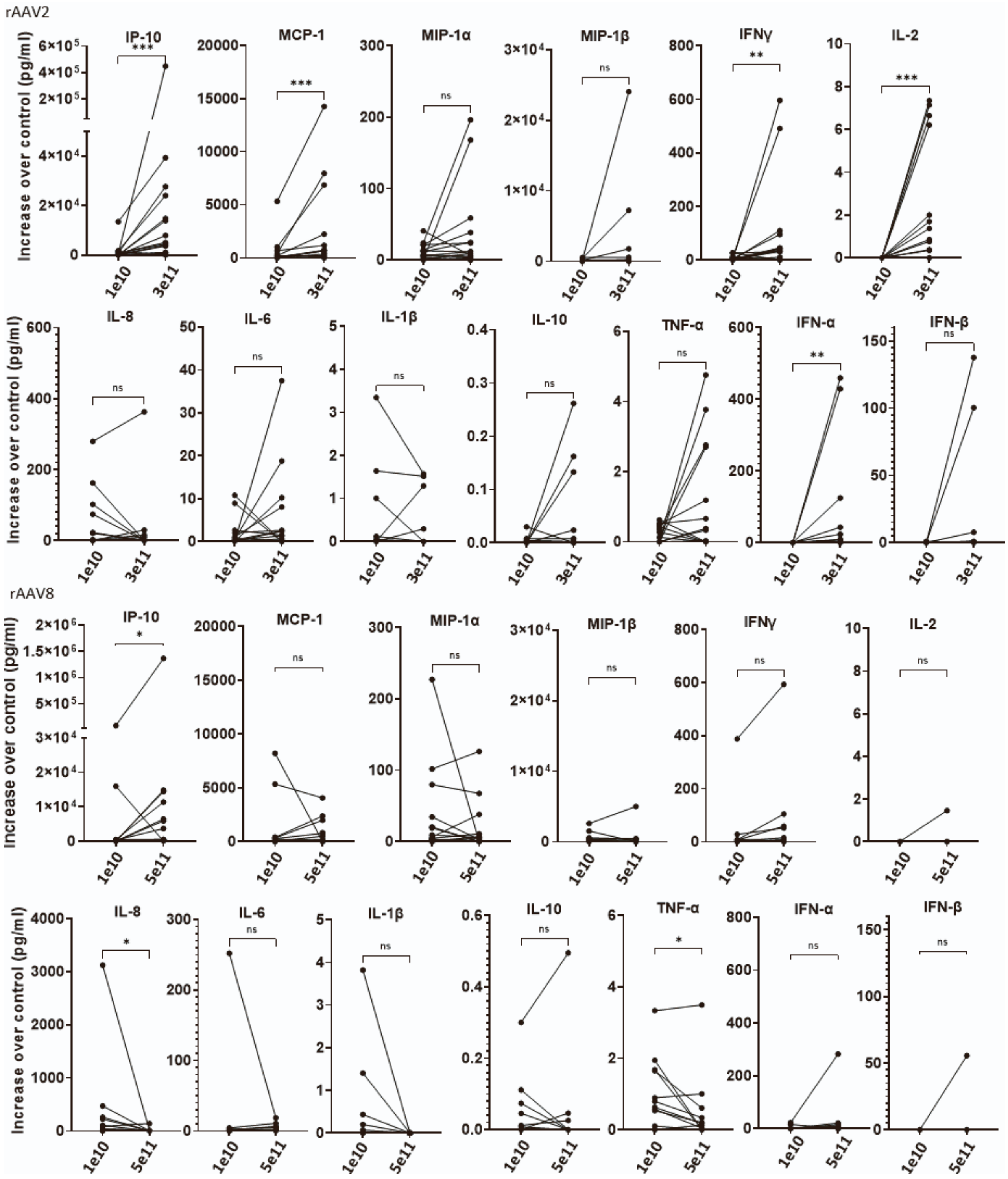

Figure S4: Healthy donor blood (n=19 donors) was incubated with different concentrations of rAAV (vg/ml). After 24 hrs, supernatants were collected for cytokine measurement. Data from four independent experiments. **(A,B)** Cytokine

concentration increases upon rAAV2 stimulation **(A)** or rAAV8 stimulation **(B)**.Significance was determined by Wilcoxon test. *$p \leq 0.05$; ** $p \leq 0.01$; *** $p \leq 0.001$; **** $p \leq 0.0001$
